# MRN-dependent and independent pathways for recruitment of TOPBP1 to DNA double-strand breaks

**DOI:** 10.1371/journal.pone.0271905

**Published:** 2022-08-02

**Authors:** Katrina Montales, Kenna Ruis, Howard Lindsay, W. Matthew Michael

**Affiliations:** 1 Department of Biological Sciences, Molecular and Computational Biology Section, University of Southern California, Los Angeles, California, United States of America; 2 Faculty of Health and Medicine, Lancaster Medical School, Lancaster University, Lancaster, United Kingdom; Tulane University Health Sciences Center, UNITED STATES

## Abstract

Ataxia Telangiectasia mutated and RAD3-related (ATR) kinase is activated by DNA replication stress and also by various forms of DNA damage, including DNA double-strand breaks (DSBs). Recruitment to sites of damage is insufficient for ATR activation as one of two known ATR activators, either topoisomerase II-binding protein (TOPBP1) or Ewing’s tumor-associated antigen 1, must also be present for signaling to initiate. Here, we employ our recently established DSB-mediated ATR activation in *X**enopus* egg extract (DMAX) system to examine how TOPBP1 is recruited to DSBs, so that it may activate ATR. We report that TOPBP1 is only transiently present at DSBs, with a half-life of less than 10 minutes. We also examined the relationship between TOPBP1 and the MRE11-RAD50-NBS1 (MRN), CtBP interacting protein (CtIP), and Ataxia Telangiectasia mutated (ATM) network of proteins. Loss of MRN prevents CtIP recruitment to DSBs, and partially inhibits TOPBP1 recruitment. Loss of CtIP has no impact on either MRN or TOPBP1 recruitment. Loss of ATM kinase activity prevents CtIP recruitment and enhances MRN and TOPBP1 recruitment. These findings demonstrate that there are MRN-dependent and independent pathways that recruit TOPBP1 to DSBs for ATR activation. Lastly, we find that both the 9-1-1 complex and MDC1 are dispensable for TOPBP1 recruitment to DSBs.

## Introduction

DNA DSBs pose a major challenge to the maintenance of genome stability. Unless they are repaired properly, DSBs can trigger cell death, or organismal death via accumulation of cancer-causing mutations. To defend themselves against DSBs cells have evolved the DNA damage checkpoint, which is organized by two protein kinases, ATM and ATR [1–6]. DSBs activate ATM and ATR, and these kinases go on to play crucial roles in promoting DNA repair and halting cell cycle progression, to allow time for repair. The means by which ATM is activated at DSBs is well understood; the MRN complex binds the free DNA end and, in turn, binds ATM in a manner that disrupts inactive ATM dimers to produce catalytically active monomers [1].

**Fig 1:**
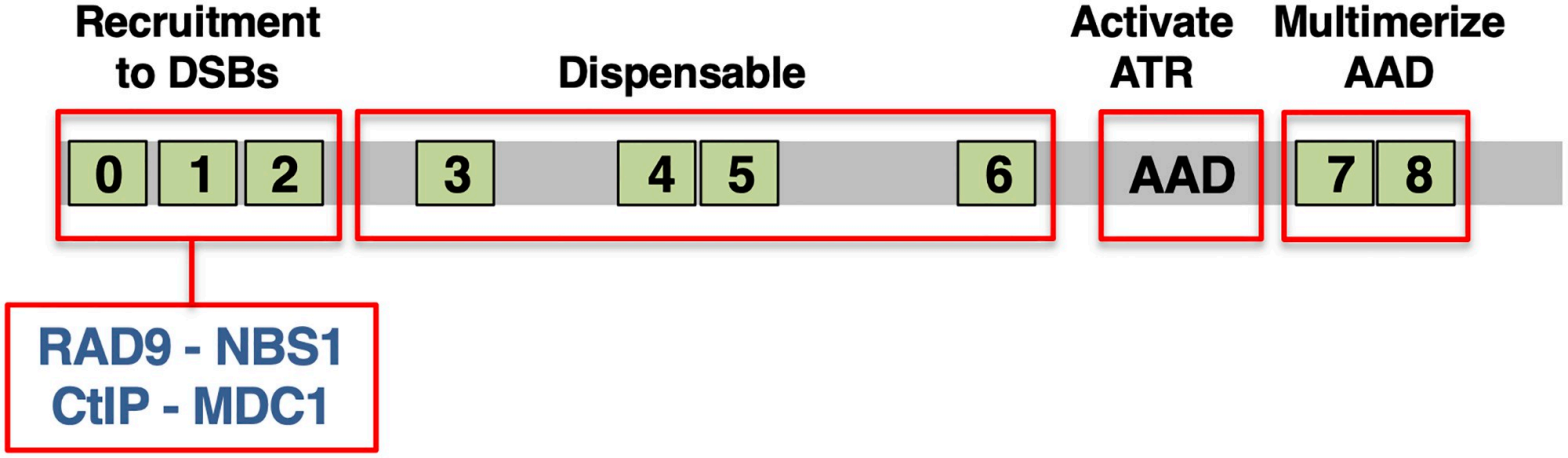
Functional domains and binding partners of TOPBP1. Please see text for details.

The mechanism for ATR activation at DSBs is far less understood. ATR signaling requires one of two known activators, either TOPBP1 or ETAA1 [7]. Recent work from our group has shown that, in *Xenopus* egg extracts (XEEs), TOPBP1 is solely responsible for ATR activation at DSBs [8,9]. TOPBP1 is a multi-functional protein with roles in ATR signaling, the initiation of DNA replication, transcriptional regulation, and DNA repair during mitosis [10]. It is comprised of nine copies of the BRCA1 C-Terminal (BRCT) domain, as well as an ATR activation domain (AAD), located between BRCT6 and BRCT7 (Fig 1). Recent work from our group has delineated a minimal, synthetic form of TOPBP1, named Junior, that is competent for ATR activation by DSBs [9]. To create Junior the sequences spanning BRCT3 to BRCT6 were removed, and it was shown that the remaining sequences are sufficient for regulated activation of ATR (Fig 1). This work also went on to show that the role for the BRCT0-2 region is to recruit TOPBP1 to DSBs, and it is dispensable thereafter, and that the role of the BRCT7&8 region is to help multimerize the AAD. AAD multimerization is essential for its ability activate ATR [11,12]. Although it is now clear that BRCT0-2 controls TOPBP1 recruitment, it remains unclear how this happens mechanistically, and thus the major goal for the current study was to biochemically delineate how TOPBP1 associates with DSBs.

To study this important problem we utilized our recently developed DMAX system, which is based on XEEs and the use of linear dsDNA templates [8,9]. For DMAX, we use the high-speed supernatant (HSS) form of XEE, where traditionally prepared crude extracts are centrifuged at high speed to produce an extract containing just soluble protein, and devoid of pigment granules, yolk platelets, and membranes. To study ATR activation we simply add phage lambda DNA that had been digested with EcoRI (to produce six fragments, sizes ranging from 3.5 kb to 21 kb) to HSS, and this results in robust activation of both ATM and ATR [8]. The system is also amendable to studying how proteins associate with DSBs. For this, we produce magnetic streptavidin beads that are linked to 5kb dsDNA molecules by virtue of a biotin group on one end of the dsDNAs. The 5kb size was chosen because linear dsDNAs that are any smaller fail to activate ATR in our system [8]. These “DSB beads” thus contain one free DNA end, which robustly activates both ATM and ATR [8,9]. Beads are added to HSS, incubation ensues, and then the beads are recovered back out of the extract, washed, and probed for occupancy of proteins of interest. Using this system, we report here that TOPBP1 can access multiple pathways for recruitment to DSBs. One pathway runs through the MRN complex, and the other pathway(s) is novel, and does not involve the known TOPBP1 interacting proteins RAD9 or MDC1. We also report that ATM kinase plays a role in limiting TOPBP1’s association with DSBs.

## Results

### Recombinant BRCT0-2 dominantly inhibits TOPBP1 from binding DSBs and reveals the transient nature of TOPBP1’s association with DSBs

**Fig 2:**
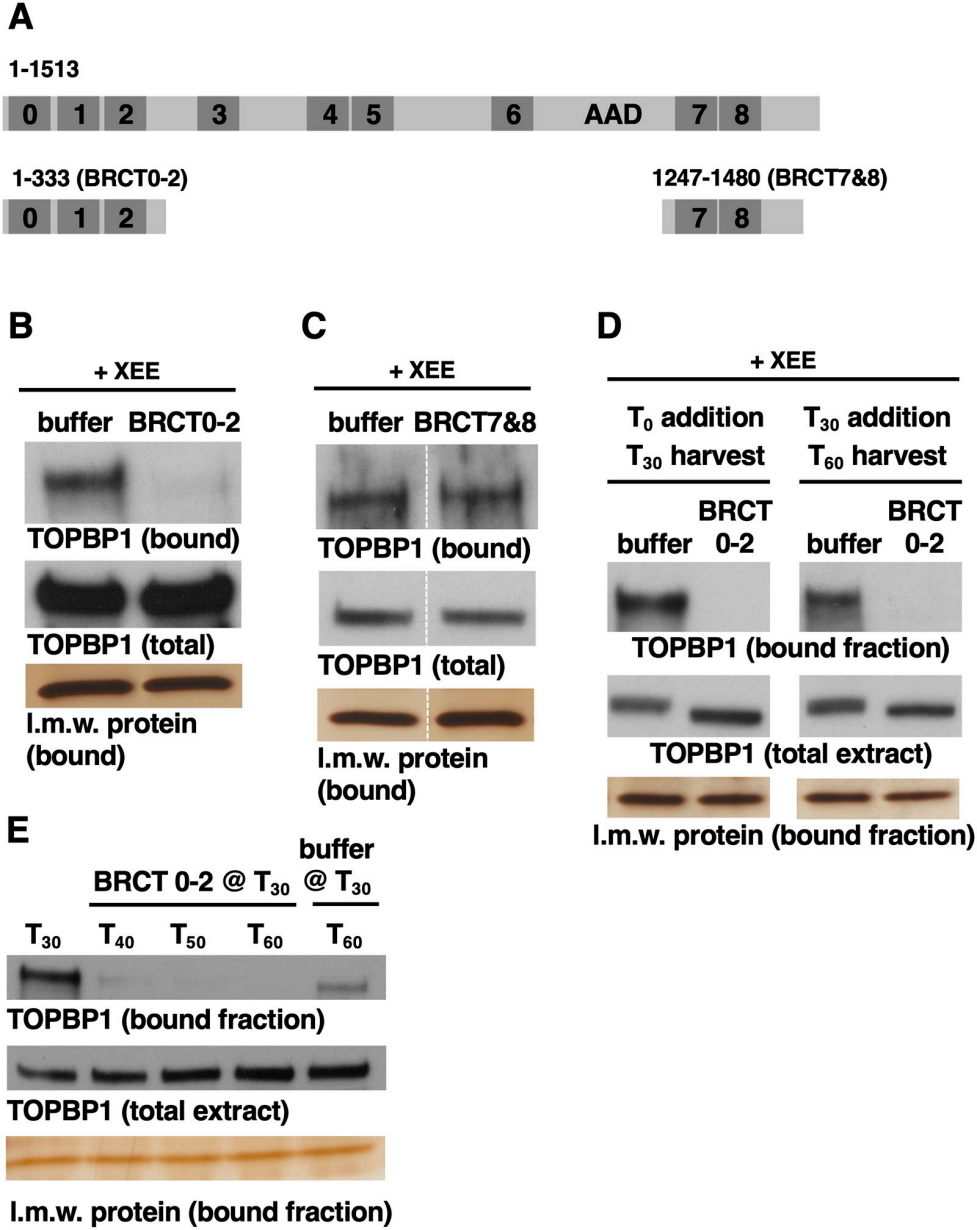
TOPBP1’s residency on DSBs is short-lived. **A.** A cartoon depicting full-length TOPBP1 and the two smaller fragments that were used in this experiment. **B.** A DSB-binding assay was performed under conditions where either buffer (PBS) or 1uM T7-6His-TOPBP1 BRCT0-2 was added to the XEE prior to addition of DSB beads. After incubation with DSB beads the beads were isolated, washed, and the samples were probed for TOPBP1. “Bound” refers to the DSB-bound material. “Total” refers to total extract, where 1 ul of the XEE used for these samples was probed for TOPBP1. The bottom panel is a silver stain gel of low molecular weight proteins bound to the DSBs, which serve as a control for equal isolation of the beads. The experiment shown is representative of two independently performed biological replicates. **C.** Same as (B) except T7-6His-TOPBP1 BRCT7&8 was used. The experiment shown is representative of two independently performed biological replicates. **D.** A DSB binding assay was performed where 1 uM T7-6His-TOPBP1 BRCT0-2 was added to the XEE either at the beginning of the experiment, at the same time as the DSB beads (T_0_ addition), or 30 minutes after addition of DSB beads (T_30_ addition). The beads were isolated at the indicated times and probed for TOPBP1 occupancy. The experiment shown is representative of two independently performed biological replicates. **E.** A DSB binding assay was performed where DSB beads were added at the beginning of the experiment (for all samples) and then isolated after 30 minutes (lane 1), 40 minutes (lane 2), 50 minutes (lane 3), or 60 minutes (lanes 4 and 5). 1 uM T7-6His-TOPBP1 BRCT0-2 was added to samples 2–4 at 30 minutes. Sample 5 received buffer (PBS) at 30 minutes. The experiment shown is representative of two independently performed biological replicates.

The major goal of this study was to leverage our DMAX system to better define how the crucial ATR activator TOPBP1 associates with DNA DSBs. Our previous work had shown that addition of a recombinant protein corresponding to the TOPBP1 BRCT0-2 region to XEE, at 1 uM (approximately 25-fold excess over endogenous TOPBP1), efficiently blocks ATR activation [8]. Furthermore, other work from our group has shown that sole function of the BRCT0-2 region is to recruit TOPBP1 to DSBs [9], and work from others has shown that human TOPBP1 uses it BRCT0-2 region to gain entry into damage-induced, sub-nuclear foci in G1 cells [13]. It thus stands to reason that excess recombinant BRCT0-2 blocks ATR activation by preventing recruitment of endogenous TOPBP1 to DSBs. To determine if this is so, we performed a DSB-binding assay, as we have done in the past [8,9]. XEE was supplemented with either buffer or 1 uM recombinant, his-tagged BRCT0-2 protein (amino acids 1–333, Fig 2A), and then DSB beads were added. After a 30-minute incubation, the beads were isolated, washed, and probed for DSB-binding proteins. In the buffer-treated sample we could readily detect TOPBP1 on the DSBs (Fig 2B), and we note that our previous work has shown clearly that TOPBP1 does not bind to beads lacking DNA [8,9]. By contrast, in samples containing recombinant BRCT0-2, there was no detectable TOPBP1 on the DSBs (Fig 2B). This effect of excess BRCT0-2 was specific, as we observed that addition of 1uM his-tagged BRCT7&8 had no impact on recruitment of TOPBP1 to DSBs (Fig 2C). We note that the blot shown in Fig 2C had one irrelevant lane removed, indicated by the dashed white lines. The full blot is displayed in S1 Fig.

Having identified recombinant BRCT0-2 as a tool to block TOPBP1 association with DSBs, we took advantage of this reagent to examine the stability of TOPBP1’s association with DSBs. More specifically, we wanted to know if TOPBP1 persists on the DSB after initial recruitment, or if its presence on DSBs is transient. For this we set up DSB-binding reactions and recombinant BRCT0-2 was added either at the beginning, or 30 minutes after the reactions had been initiated. The rationale is if TOPBP1 stably associates with DSBs then BRCT0-2 would be able to prevent TOPBP1 from binding DSBs when added at the beginning of the reaction, but it would be less able to block binding when added at the 30-minute timepoint. As shown in Fig 2D, recombinant BRCT0-2 could potently prevent TOPBP1 from binding DSBs when added at T_0_, and, surprisingly, it also prevented TOPBP1 accumulation at DSBs when added at T_30_. Thus all of the TOPBP1 that was bound to DSBs during the first 30 minutes had become disassociated from the DNA over the ensuing 30 minutes when BRCT0-2 was present. To pursue these observations we narrowed the time window for incubation of DSB beads with excess BRCT0-2, and found that in as little as 10 minutes nearly all of the TOPBP1 on the DSBs had disassociated from the DNA (Fig 2E). Based on these data, we conclude that active TOPBP1-ATR signaling complexes are only transiently present on DSBs, and thus to sustain signaling new complexes are constantly forming as older ones disassemble from the DNA.

### MRN is partially required for TOPBP1 recruitment to DSBs

**Fig 3:**
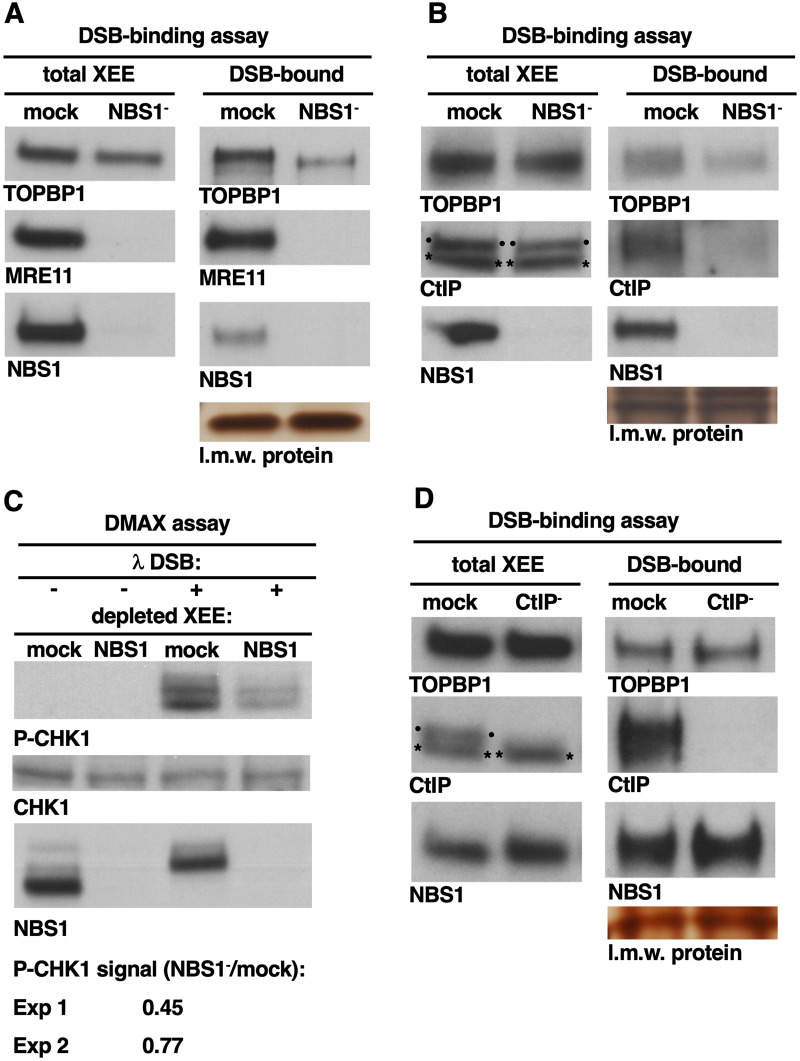
MRN is partially required for TOPBP1 recruitment to DSBs and ATR signaling. **A.** NBS1 antibodies were used to remove the MRN complex from XEE via immunodepletion. A mock-depleted sample was also prepared. The depleted extracts were then probed for the indicated proteins (panels “total XEE”). Note that NBS1 depletion results in the removal of all detectable NBS1, as well as MRE11, showing that the MRN complex has been removed. Note also that TOPBP1 is not co-depleted by the NBS1 antibody. The depleted extracts were then used for a DSB binding assay, and panels “DSB-bound” shows the occupancy of the indicated proteins on the DSB beads. The experiment shown is representative of two independently performed biological replicates. **B.** Same as (A) except CtIP occupancy was examined in this experiment. The experiment shown is representative of two independently performed biological replicates. **C.** NBS1 antibodies were used to remove the MRN complex from XEE via immunodepletion. A mock-depleted sample was also prepared. The depleted extracts were then used for a DMAX assay to asses ATR’s ability to phosphorylate its key substrate, CHK1. “λ DSBs”refers to phage lambda DNA that was digested with EcoRI and represents the source of DSBs for this experiment. DSBs were optionally added to the depleted extracts, and after incubation samples were pulled and probed for P-CHK1, to assess the ATR activity state, and unmodified CHK1, to ensure CHK1 was present in the extract, and NBS1, to assess the effectiveness of the immunodepletion. The experiment shown is representative of two independently performed biological replicates. The other replicate is shown in S2 Fig. Shown below the blots is quantification of P-CHK1. For both replicates, ImageJ software was used to quantify the signal intensity for the P-CHK1 samples. The values for the mock-depleted sample were set to 100, the values for the NBS1-depleted samples were adjusted accordingly, and the results are presented as a ratio of NBS1-depelted signal over mock-depleted signal. “Exp” stands for experiment. **D.** Same as (A) except CtIP was depleted in this experiment. The experiment shown is representative of two independently performed biological replicates. The CtIP antibody we used recognizes a background band in the total extract, that runs just below CtIP. The background band is denoted by two asterisks while the CtIP band is denoted by two dots. Note that the background band remains after CtIP depletion whereas all detectable CtIP is removed.

Our recent work has shown that, for DSB recruitment, TOPBP1 uses its BRCT0-2 domains to bind a factor present at the DSB [9]. The identity of this factor is not known, and thus to examine this we took a candidate approach, and we began with the MRN/ATM/CtIP network of proteins as possible mediators of TOPBP1 recruitment to DSBs. All three of these factors have been implicated in TOPBP1 function at DSBs in the past. MRN has been shown to recruit Xenopus TOPBP1 to a circular, ssDNA structure containing primers annealed to it [14]. In human cells, the NBS1 subunit of MRN has been implicated in ATR activation on resected DNA [15], and in XEEs MRN is required for TOPBP1 to activate ATR when the AT70 DNA structure is used as the source of DNA [16]. Neither the circular, ssDNA structure containing primers nor the AT70 structure actually resemble a DSB, and thus it was an open question if MRN regulates TOPBP1 at DSBs. To address this we combined immunodepletion of MRN with DSB binding assays. Antibodies against NBS1 [17] were used to remove MRN (Fig 3A, panel “total XEE”). Blots of the total extract were probed for NBS1, MRE11, and TOPBP1, and these data show that neither NBS1 nor MRE11 were detectable after depletion, however TOPBP1 was present in the NBS1-depleted extract. These extracts were then used in a DSB-binding assay, and we observed that the level of TOPBP1 on DSBs in the NBS1-depleted extract was reduced, but not eliminated, relative to the mock-depleted control (Fig 3A, panel “DSB-bound”). This reduction in TOPBP1’s occupancy on DSBs after MRN removal was highly reproducible across multiple experiments (see below). Thus, MRN is needed for efficient recruitment of TOPBP1 to DSBs, but it is not absolutely essential. We note that multiple attempts at purification of active MRN complex were all unsuccessful and thus we were unable to perform an add-back experiment. We next determined if MRN is required to recruit CtIP to DSBs in our system, as has been reported for other systems [18,19]. MRN was depleted from XEE (Fig 3B, panel “total XEE”), and DSB-binding was again assessed. As shown in Fig 3B, panel “DSB-bound”, loss of MRN again diminished, but did not eliminate, TOPBP1’s recruitment. For the experiments shown in Fig 3A and 3B, quantification of the TOPBP1 signals in the DSB bound samples show that loss of MRN results in a 60.8% and 50.6% reduction in DSB occupancy, respectively. By contrast, CtIP recruitment was severely attenuated in the MRN-depleted extract, as expected (Fig 3B). Thus, while CtIP has an absolute dependence on MRN for binding to DSBs, TOPBP1 has only a partial dependence. We note that it is formally possible that a small amount of MRN, undetectable by Western blot, may still be present in our MRN-depleted extract and that this small amount is sufficient to recruit TOPBP1 to DSBs. We feel this is unlikely, however, given that our depletion conditions result in a severe loss of DSB-bound CtIP, strongly suggesting that the extract is indeed functionally depleted of MRN.

Our findings demonstrate that MRN plays a role in TOPBP1 recruitment to DSBs, however when MRN is absent TOPBP1 can nonetheless gain access to DSBs. This raises the important question of whether the TOPBP1 present on DSBs in MRN-depleted extracts is capable of activating ATR, or if it is performing an ATR-independent function. To answer this question, we assessed ATR activation in NBS1-depleted extract using our previously described DMAX assay [8,9]. This assay relies on the use of EcoRI-digested lambda DNA (“λ DSBs”) to activate ATR, which is assessed by probing for the Serine 345-phosphorylated form of the key ATR substrate CHK1 (S345-phosphorylated CHK1 is referred to here as P-CHK1. As shown in Fig 3D, the amount of P-CHK1 present in the NBS1-depleted sample was less than that in the mock-depleted sample, and quantification revealed a 55% reduction. This reduction varied across replicate experiments, and for the replicate shown in S2 Fig the reduction was 23%. These data show that while ATR signaling is slightly diminished after loss of MRN, it is clearly still happening. We conclude that MRN is not absolutely essential for ATR activation in our DMAX system.

### ATM limits the amount of TOPBP1 that associates with DSBs

**Fig 4:**
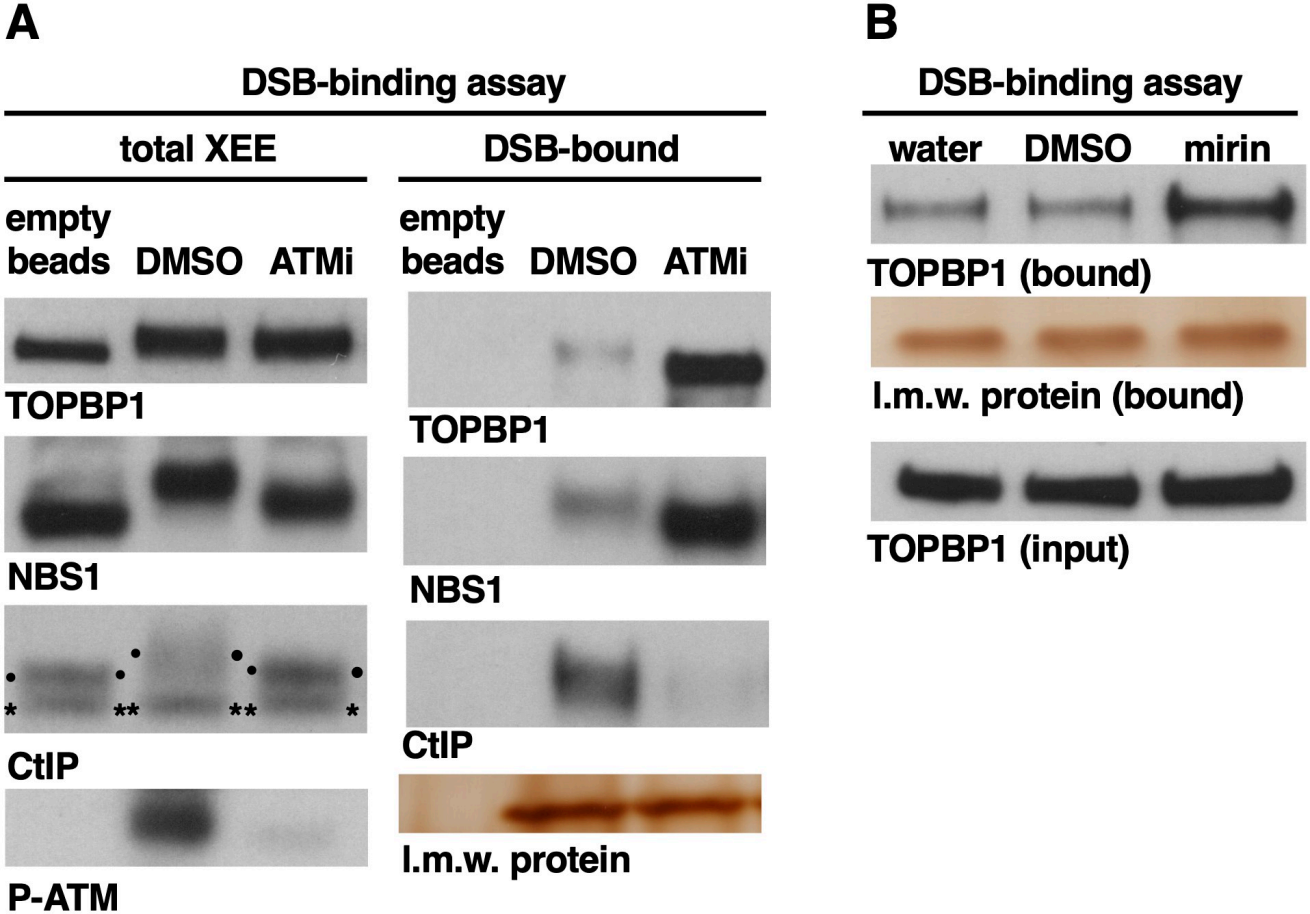
Loss of ATM kinase activity promotes the accumulation of MRN and TOPBP1 on DSBs. **A.** A DSB binding assay was performed. One sample received magnetic streptavidin beads lacking DNA (empty beads) and the other two samples received DSB beads. Beads were incubated in XEEs treated with the indicated chemicals. ATMi is KU55933 at 100 uM. After incubation, samples were taken of the total extract and probed for the indicated proteins (panels “total XEE”). Please see the legend for Fig 3D for an explanation of the CtIP panel. Beads were isolated and washed and the probed for occupancy of the indicated proteins (panels “DSB-bound”). The experiment shown is representative of two independently performed biological replicates. **B.** A DSB-binding assay was performed in XEEs treated with the indicated compounds. Mirin was used at 100 uM. The experiment shown is representative of two independently performed biological replicates.

We next turned to ATM. ATM has been shown to play an important role in ATR activation, in both Xenopus and cultured human cells [20–22], however it was not previously known if ATM is needed to recruit TOPBP1 to DSBs. To explore this we employed a small molecule inhibitor of ATM, KU55933 (ATMi), and added it, or a vehicle-only control (DMSO), to XEE. DSB beads were then added and, after incubation, the beads were recovered and probed for the presence of TOPBP1, NBS1, and CtIP. As shown in Fig 4A, none of the factors bound to empty beads, and all three were found on DSB beads in the control, DMSO-treated sample. In the ATMi-treated sample, we found that the levels of both TOPBP1 and NBS1 were elevated on the DSB beads, relative to the control, whereas CtIP occupancy on DSB beads was dramatically reduced, as expected based on previous findings that ATM kinase activity promotes CtIP recruitment [18]. To query the efficacy of ATMi in this experiment we probed total extract for auto-phosphorylation of ATM’s serine 1981 (P-ATM), a hallmark of ATM activity [23], and found that while P-ATM was induced by DSBs in the vehicle-treated sample, the ATMi-treated sample was devoid of detectable P-ATM. Thus, ATM kinase activity is not required for TOPBP1 accumulation at DSBs, and in its absence TOPBP1 appears to hyper-accumulate, as does NBS1. To confirm this result we used an independent inhibitor, mirin, which targets MRE11 and effectively blocks ATM activation in XEEs [24]. Like ATMi, mirin promoted the accumulation of TOPBP1 on DSBs, relative to controls (Fig 4B).

**Fig 5:**
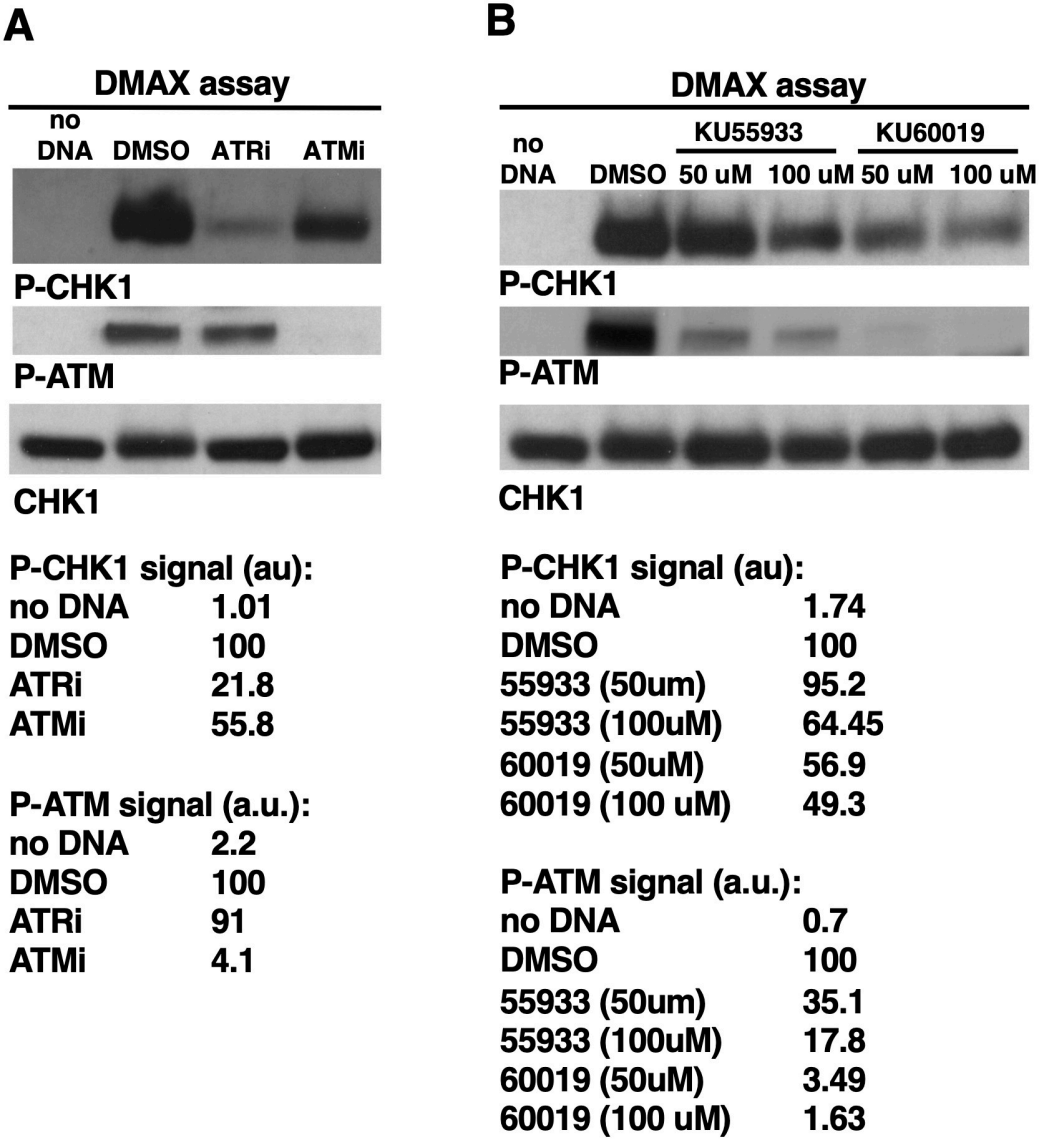
ATM kinase activity is not essential for ATR signaling in the DMAX system. **A.** XEEs were treated with the indicated compounds and then λ DSBs were optionally added. After incubation samples were taken and probed for the indicated proteins. The P-CHK1 and P-ATM signals were then quantified, as in Fig 3C. ATRi was present at 100 uM, as was ATMi (KU55933). The experiment shown is representative of two independently performed biological replicates. **B.** Same as (A) except ATRi was omitted and two different ATMis were used, at the indicated concentrations. The experiment shown is representative of two independently performed biological replicates.

Data shown thus far demonstrate that loss of MRN attenuates, but does not eliminate, TOPBP1’s recruitment to DSBs and subsequent activation of ATR. Previous work has shown that MRN is required for ATM activation, and thus our data suggest that, like MRN, ATM is not absolutely essential for ATR activation in our DMAX system. To address this directly, we examined ATR signaling in XEEs that were treated with ATMi (KU55933 at 100uM)). ATMi had a modest effect on ATR signaling, whereas inclusion of ATRi had a much more robust impact on P-CHK1 (Fig 5A, ATRi reduces P-CHK1 by 78.2% compared to 44.2% for ATMi). Importantly, ATMi did have a strong impact on P-ATM (95.9% reduction), whereas ATRi did not (just a 9% reduction). To confirm this result we used a different ATMi, this one termed KU60019, and again we observed that ATMi could dramatically reduce the P-ATM signal, but the P-CHK1 signal was only modestly reduced (Fig 5B, see quantification below the blots). These data are consistent with the effect of depleting MRN on ATR signaling (Fig 3C and S2 Fig). We conclude that ATM promotes ATR signaling, but it is not essential for ATR signaling. One issue that arises from these data is that while ATMi reduces ATR signaling, it increases the amount of both MRN and TOPBP1 present on the DNA (Figs 4A, 5A and 5B). How can signaling go down when the amount of TOPBP1 on the DNA goes up? MRN is also clearly increased on DNA by ATMi, and given that MRN recruits TOPBP1 to DSBs, we can understand why TOPBP1 levels also go up. One plausible explanation for the ATR signaling profile is that the TOPBP1 that is recruited via MRN requires ATM in order to activate ATR, and thus despite having more MRN-recruited TOPBP1 on DNA this TOPBP1 is inactive and ATR signaling is thus limited to the TOPBP1 that is recruited independently of MRN. Why blocking ATM results in an increase in MRN on DNA is an interesting question for future research.

### DNA structures can distinguish the MRN-dependent and independent pathways for ATR activation

**Fig 6:**
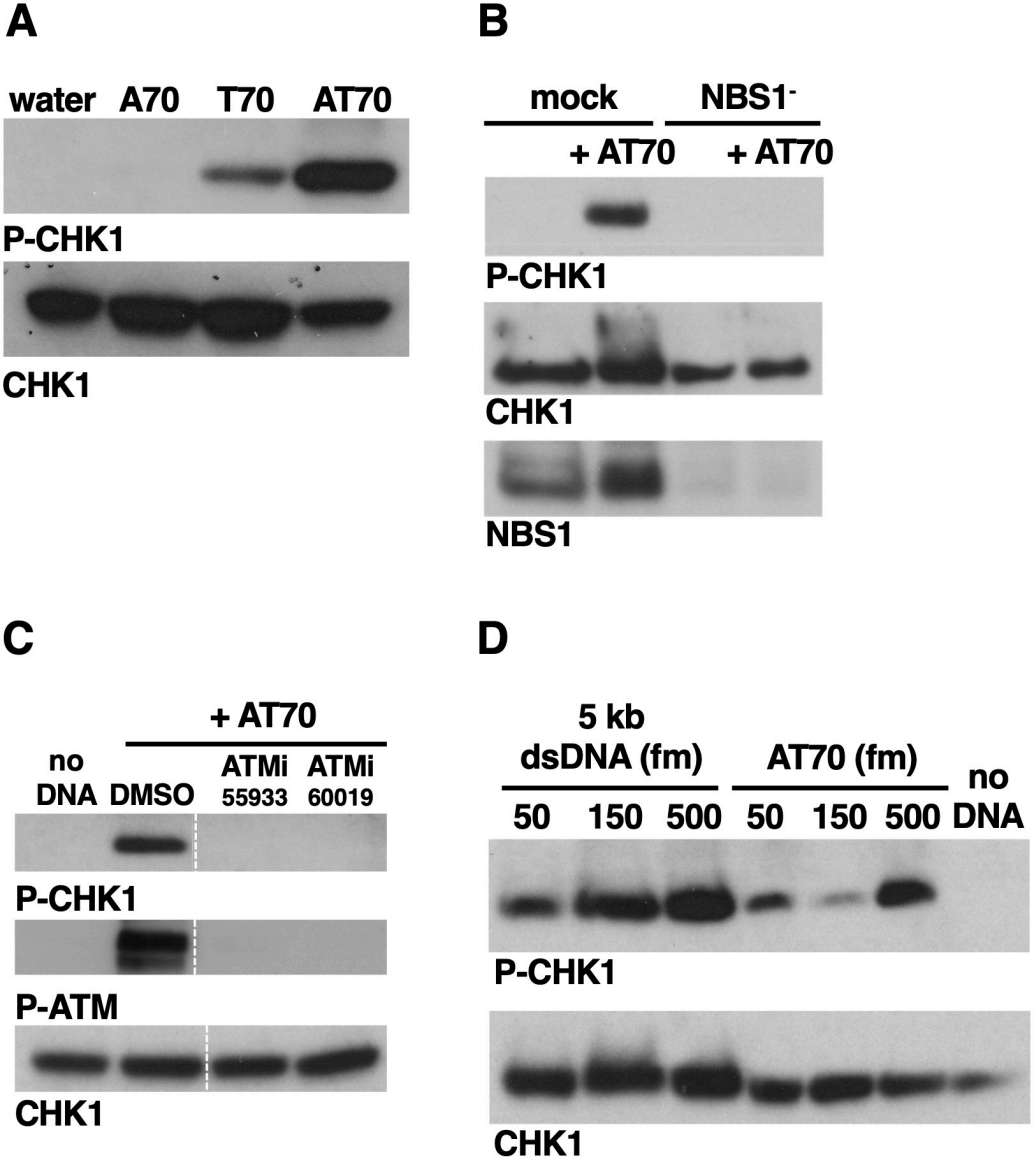
The AT70 DNA structure requires MRN and ATM for ATR signaling. **A.** XEEs were incubated with either water, the A70 oligonucleotide, the T70 oligonucleotide, or the two oligonucleotides that had been annealed together (AT70). The samples were then probed for P-CHK1 and CHK1. The experiment shown is representative of two independently performed biological replicates. **B.** AT70 was optionally added to either mock- or NBS1-depleted extract. After incubation, samples were pulled and probed for the indicated proteins. The experiment shown is representative of two independently performed biological replicates. **C.** Extracts were treated with the indicated compounds and AT70 was optionally added. After incubation, samples were pulled and probed for the indicated proteins. ATMis were present at 100 uM. The experiment shown is representative of two independently performed biological replicates. **D.** The indicated DNAs at the indicated concentrations were added to XEEs and, after incubation, samples were pulled and probed for the indicated proteins. The experiment shown is representative of two independently performed biological replicates.

Our data show that both MRN-dependent and MRN-independent pathways can recruit TOPBP1 to DSBs for ATR activation. These findings are seemingly at odds with previous work that used the AT70 DNA structure [25] to activate ATR in unfractionated XEE (low-speed supernatant or LSS), as these studies showed that MRN is essential for ATR activation by AT70 [16]. To reconcile our data with these previously published data, we examined the requirements for AT70 activation of ATR in our HSS-based DMAX system. AT70 is formed via annealing of two homopolymeric 70mers, poly-A (A70) and poly-T (T70). We found that A70 could not activate ATR and that T70 could, albeit at a lessor efficiency than AT70 (Fig 6A). These results are in good agreement with previous studies [25], and thus AT70 can activate ATR in both the HSS and LSS extract systems. We next asked if MRN is required for AT70 mediated ATR activation in HSS, as it is in LSS [16]. MRN was removed via immunodepletion and, as shown in Fig 6B, loss of MRN prevented ATR activation by AT70. Thus MRN is essential for ATR activation by AT70 in our HSS-based system. We also asked if AT70-mediated ATR activation is sensitive to ATM inhibitors. As shown in Fig 6C, both ATMis could block ATR activation. Thus, for AT70, the requirements for MRN and ATM in ATR activation are much more stringent than they are for DSBs. We next compared the ability of dsDNA and AT70 to activate ATR when present at equimolar concentrations, and observed that, across a 10-fold concentration range, the dsDNA (in this case a 5kb PCR fragment) consistently activated ATR more robustly than did AT70 (Fig 6D). These data show that different DNA structures can access different pathways for ATR activation–dsDNA can access both the MRN/ATM-dependent and independent pathways, whereas AT70 can only access the MRN/ATM-dependent pathway. Accordingly, dsDNA is the more potent ATR activator. We note that how AT70 activates ATR is unclear. Our previous work has shown that linear, dsDNAs must be of at least 5kb in length for robust ATR activation in our extracts, and that dsDNAs of less than 3kb do not activate ATR at all [8]. Thus why the considerably smaller AT70 can activate ATR is a mystery. Another conundrum surrounding AT70 is that its GC70 counterpart cannot activate ATR [25]. Thus there is something clearly unique about how AT70 interfaces with the ATR machinery, however the physiological relevance of AT70 is not yet clear.

### Both the 9-1-1 complex and MDC1 are dispensable for TOPBP1 recruitment to DSBs

**Fig 7:**
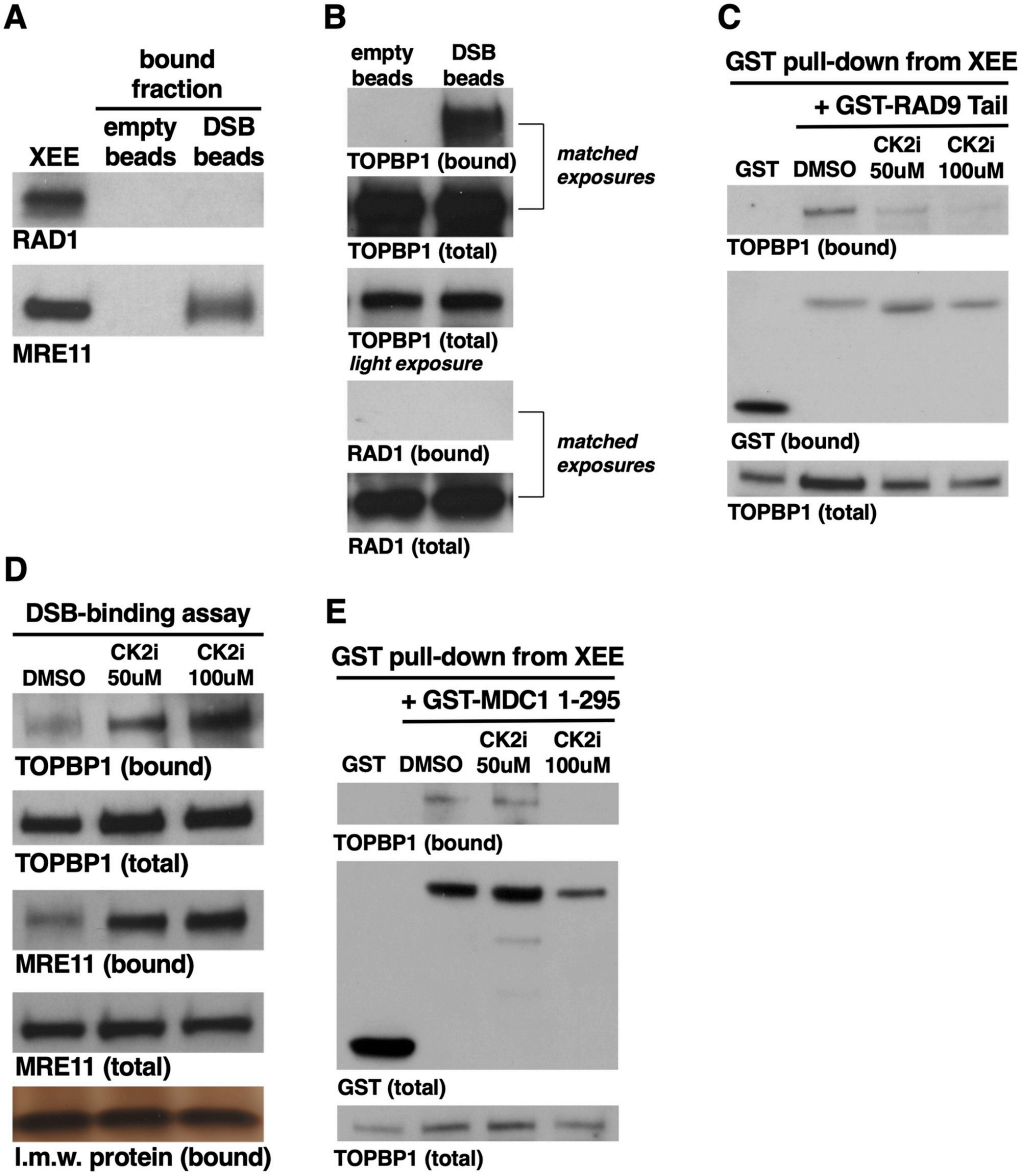
Neither the 9-1-1 complex nor MDC1 are required for TOPBP1 recruitment to DSBs. **A.** A DSB binding assay was performed and the bound fraction was probed for the indicated proteins. The experiment shown is representative of two independently performed biological replicates. **B.** A DSB binding assay was performed and samples from total extract and the bound fraction was probed for the indicated proteins. For the total extract samples, 1 ul of XEE was loaded on the gel. Please see text for explanation of “matched exposures”. The experiment shown is representative of two independently performed biological replicates. **C.** A GST pull-down experiment was performed. The indicated GST fusion proteins were added to XEEs that had been treated with the indicated compounds and, after incubation, complexes were isolated back out of the extract via glutathione-sepharose beads and probed for the indicated proteins. Samples of the total extract were also probed for TOPBP1. The experiment shown is representative of two independently performed biological replicates. **C.** A DSB-binding assay was performed with XEEs treated with the indicated compounds at the indicated concentrations. Total extract and DSB-bound samples were then probed for the indicated proteins. The experiment shown is representative of two independently performed biological replicates. **D.** Same as (C) except a GST-MDC1 fusion protein was used in place of GST-RAD9 Tail. The experiment shown is representative of two independently performed biological replicates.

Data reported here reveal an MRN-independent pathway for TOPBP1 recruitment to DSBs and ATR activation. Which factors might mediate this? Over the years, many different proteins have been shown to bind TOPBP1’s recruitment domain, the BRCT0-2 region, and those with known or suspected roles in DSB repair include RAD9 of the 9-1-1 complex [26,27], NBS1 [16], CtIP [28], 53BP1 [29], MDC1 [30–32], and RHINO [33]. We have already examined NBS1 and CtIP, and of the remainder we could eliminate 53BP1 as a candidate as it also requires TOPBP1’s BRCT5 for binding [29], and in our DMAX system BRCT5 is dispensable for ATR activation [9]. We could also eliminate RHINO, as it is not present in the Xenopus egg, [34] and data not shown. This leaves 9-1-1 and MDC1 (Fig 1), and we initially focused on the 9-1-1 complex. We first asked if 9-1-1 associates with DSBs in our system. This was an open question, as while yeast 9-1-1 has a demonstrated role in DSB repair [35,36], whether this function is conserved in vertebrates is unclear. We performed a DSB binding assay and observed that while MRE11 is easily detectable on the DSB beads, but not empty beads lacking DNA, the RAD1 subunit of 9-1-1 was not detectable on either empty or DSB beads (Fig 7A). This finding suggests that 9-1-1 does not bind DSBs in our system, but to be certain of this we performed another DSB binding assay, and for this we directly compared the amount of either TOPBP1 or RAD1 that is present in the extract to the amount that is found on DSB beads. This was done by running the total extract samples and DSB-bound fractions on the same gel, side-by-side, and then exposing the blots for the same amount of time (hence “matched exposures”). As shown in Fig 7B, the amount of TOPBP1 captured on DSB beads from a binding reaction containing 7 ul of XEE is just under the amount present in 1ul of total extract, suggesting that a little less than 14% of the available TOPBP1 was bound to the beads. By stark contrast, and even after prolonged exposure of the blot, we could not detect any 9-1-1 complex on the DSB beads (Fig 7B). These data reveal that 9-1-1 and TOPBP1 are not stoichiometrically associated with one another on DSBs, however, it may be that 9-1-1 is only transiently associated with DSBs but nonetheless responsible for recruiting TOPBP1. To pursue this possibility, we next asked if blocking the TOPBP1-RAD9 interaction would impact TOPBP1’s ability to bind DSBs. CK2 phosphorylation of the RAD9 tail domain is required for binding to TOPBP1 [37], and we have previously shown that the CK2 inhibitor CX-4945 prevents the TOPBP1-RAD9 interaction in XEE [11]. Indeed, when CK2i is titrated into XEE it prevents interaction between endogenous TOPBP1 and the RAD9 tail in a GST pull-down assay (Fig 7C). By contrast, CK2i did not hamper TOPBP1’s ability to bind DSBs, and it actually increased binding (Fig 7D). These data make it clear that the interaction between RAD9 of 9-1-1 and TOPBP1 is dispensable for TOPBP1 recruitment to DSBs. These data also show that MDC1, which also relies on CK2-mediated phosphorylation to bind TOPBP1 [32,33], is not involved in recruiting TOPBP1 to DSBs. To confirm this we asked if CK2i would block the MDC1-TOPBP1 interaction in XEEs, and found that it could (Fig 7E). We conclude that neither 9-1-1 nor MDC1 play a role in TOPBP1 recruitment, and thus future work will be focused on identifying the factor(s) that recruits TOPBP1 for the MRN-independent pathway. By extension, we can also eliminate HTATSF1, which also interacts with TOPBP1 in a CK2-dependent manner [38].

## Discussion

**Fig 8:**
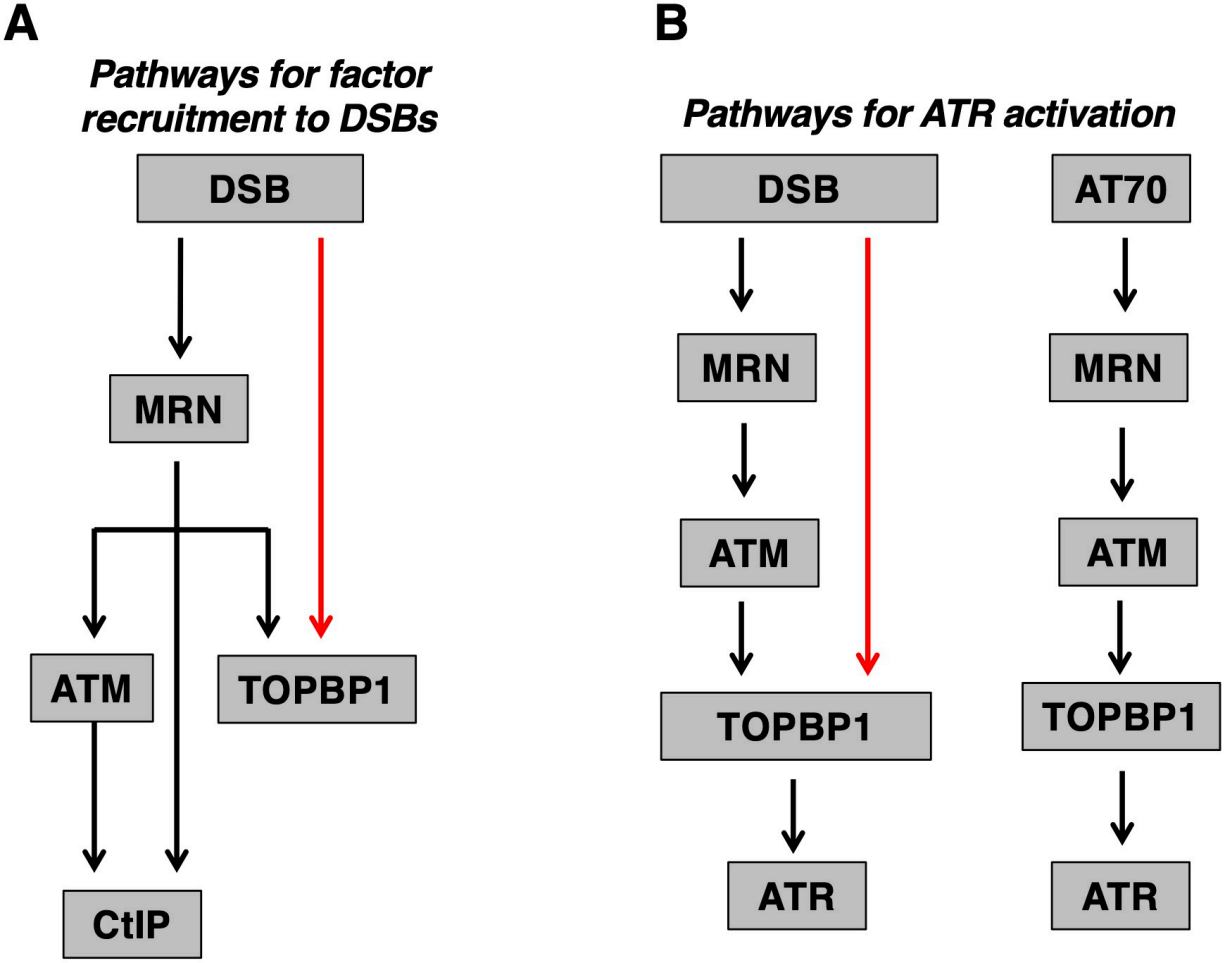
Multiple pathways for TOPBP1 recruitment and ATR activation by DSBs in *Xenopus*. **A.** Please see text for details. **B.** Please see text for details.

In this study we analyzed how TOPBP1 and the MRN/ATM/CtIP network of proteins are recruited to DSBs. Using our DSB bead-binding assay we found that MRN is required for CtIP recruitment (Fig 3B), as is ATM kinase activity (Fig 4A). These results are in excellent agreement with work done in mammalian and yeast systems [18,19], and they validate our binding assay as physiologically relevant to the study of DSB recruitment. We also found that MRN is partially required for TOPBP1 to access DSBs. For the experiments shown in Fig 3A and 3B, quantification of the TOPBP1 signals in the DSB bound samples show that loss of MRN results in a 60.8% and 50.6% reduction in DSB occupancy, respectively. Thus about half of the TOPBP1 on DSBs is recruited via an MRN-dependent pathway, and the other half via an alternative pathway (summarized in Fig 8A). We also examined the CtIP and ATM kinase requirements for TOPBP1, and we found that loss of CtIP has no impact on TOPBP1 recruitment (Fig 3D), and that loss of ATM kinase activity, using two mechanistically distinct inhibitors, actually increases TOPBP1 occupancy at DSBs (Fig 4A and 4B). Taken together, these data delineate an ordered assembly pathway where MRN controls recruitment of all of the CtIP and half of the TOPBP1 present at DSBs. MRN also activates ATM [1], which, in turn, is needed for CtIP recruitment, but not that of TOPBP1 (Fig 8A). We note that loss of ATM activity also causes an increase in MRN levels on DSBs (Fig 4A), and this likely explains the elevated levels of TOPBP1, given the requirement for MRN in TOPBP1 recruitment. A partial requirement for MRN has also been observed for TOPBP1 recruitment to stalled replication forks in XEEs [39]. In these studies it was shown that the 9-1-1 clamp loader RAD17 and MRN are both required for full TOPBP1 occupancy at stalled forks. Data shown here, however, reveal that 9-1-1 plays no role in TOPBP1 recruitment to DSBs (Fig 6), and thus if RAD17 plays a role in TOPBP1 recruitment to DSBs it would be a 9-1-1 independent role, and this seems unlikely. Further work is clearly needed to identify the components for the MRN-independent recruitment pathway.

Beyond TOPBP1 recruitment, it was also important to determine if MRN is required for ATR signaling in our system, as it was formally possible that the residual TOPBP1 on DSBs in MRN-depleted extract was irrelevant to ATR. We found a modest attenuation in P-CHK1 signal after loss of MRN (Fig 3C), consistent with the reduced DSB occupancy of TOPBP1. In addition, we found that loss of ATM kinase activity resulted in attenuated, but still active, ATR signaling (Fig 5). These data make it clear that an MRN-independent pathway not only recruits TOPBP1 to DSBs, but does so in a manner where TOPBP1 activates ATR (Fig 8B). Interestingly, we also observed that an alternative DNA structure, AT70, is absolutely dependent on both MRN and ATM for ATR activation. One explanation for this is that the MRN/ATM branch of the ATR activation pathway is specific for 5’-DNA junctions, which are likely to be present with AT70, and also after processing of linear dsDNAs. Indeed, previous work had shown that circular ssDNA containing 5’-DNA junctions activates ATR in an MRN-dependent manner [14]. Furthermore, work using human cell extracts has shown that the MRN subunit NBS1 is required for ATR signaling when DNA substrates containing 5’-DNA junctions are used to initiate signaling [15]. Thus, if we assign MRN and ATM to 5’-DNA junctions, then an important research question becomes what is the DNA structure that allows ATR activation via the MRN-independent pathway? Future work will resolve this important question.

Previous work using human cells and cell extracts has proposed a “hand-off” model for ATR activation at DSBs, where ATM is activated by MRN bound to DNA ends, and then resection ensues, and this produces the ssDNA that activates ATR [40]. Meanwhile, resection is proposed to inactivate ATM. The hand-off model thus proposes that DNA end resection is crucial for ATR activation by DSBs. Our data in *Xenopus* suggest that end resection may not be a universal requirement for ATR signaling at DSBs. In *Xenopus*, it has been shown using an identical extract system and linear, 5.7 kb dsDNAs, that MRN is required to initiate DNA end resection [41]. Thus, in our MRN-depleted extracts, resection may be blocked, yet ATR signaling still occurs. This raises the fascinating possibility that ATR can be activated by dsDNA ends, in addition to ssDNA, and experiments to test this hypothesis are in progress.

Lastly, it is important to reconcile data shown here with data from human cells showing that the accumulation of TOPBP1 into sub-nuclear, DSB-induced foci is cell cycle regulated and relies on interactions with MDC1 and 53BP1 in G1 or M phase cells [13,29,32]. Our egg extracts are treated with cycloheximide, which locks them into interphase and thus an S/G2-like state, because translation of the cyclins that drive mitotic entry is blocked. This is likely why we observe no requirements for MDC1 or 53BP1 in our system. It is important to note, however, that using incorporation into foci as a measure for recruitment to DSBs has become complicated by the recent discovery that TOPBP1 forms condensates [42]. These studies suggest that TOPBP1 is recruited to DSBs where it activates ATR. ATR, in turn, drives condensate/foci formation by TOPBP1, and this promotes an amplification of ATR signaling. Thus it may well be that initial recruitment of TOPBP1 to DSBs occurs independently of MDC1/53BP1, and that these factors are needed for the condensate/foci formation that occurs later in the process.

## Materials and methods

### Materials

### Recombinant proteins

T7-6His-TOPBP1 BRCT0-2 and T7-6His-TOPBP1 BRCT7&8 have been previously described [8]. GST-RAD9Tail has been previously described [11]. GST-MDC1 1–295 was expressed and purified from *E*. *coli* using standard procedures.

#### Antibodies

We used the following commercially sourced antibodies in this work: GST (Millipore Sigma #05–782), CHK1 (Santa Cruz Biotechnology #sc-8408), P-CHK1 (Cell Signaling Technology #2341S), and P-ATM (ATM phospho S1981 Antibody Rockland # 200-301-400S). We also used our own antibodies against *Xenopus* TOPBP1 [43] and *Xenopus* NBS1 [17]. In addition, we were gifted the following antibodies from academic laboratories: *Xenopus* RAD1 (kind gift of Karlene Cimprich, Stanford University) and *Xenopus* CtIP (kind gift of Zhongseng You, Washington University in St. Louis).

#### Chemical inhibitors

We used the following commercially sourced chemical inhibitors: ATMi (KU55933 and KU60019, both purchased from Selleckchem.com), mirin (purchased from Sigma), and CK2i (CX-4945; purchased Selleckchem.com).

### Methods

#### Xenopus husbandry

All procedures have been approved by the USC Institutional Animal Care and Use Committee under active protocol #11475. Approval date: 7/15/2021, expiration date: 7/15/2024, annual renewal date: 7/15/2022.

#### Xenopus egg extracts and immunodepletion

The high-speed supernatant of XEE was used exclusively in this study. HSS was prepared exactly as described [44]. For immunodepletion of NBS1 the procedure was performed exactly as described for depletion of TOPBP1 in [43].

#### DSB binding assay

All DSB binding assays containing XEE were performed exactly as described [8,9]. Briefly, a 5kb PCR fragment was biotinylated via inclusion of a biotin moiety on the forward PCR primer. PCR was performed with Phusion^®^ High-Fidelity DNA Polymerase (New England Biolabs), which produces blunt DNA ends. After PCR and cleanup, the 5kb PCR fragment was coupled to magnetic streptavidin beads (Dynabeads M-270 Streptavidin, ThermoFisher) according to the manufacturer’s instructions. These “DSB beads”, containing 600 fmol of dsDNA per assay, were then incubated in 20ul of HSS. After incubation the beads were collected on a magnetic stand and washed three times in PBS+0.1% TritionX-100. Bound proteins were then eluted with 2X SDS-PAGE sample buffer and examined by Western blotting.

#### DSB-mediated ATR activation in XEE (DMAX) assay

For DMAX assays, okadaic acid was first mixed with 20 μL of HSS to a final concentration of 1 μM, as described [8]. Linear dsDNA derived from EcoRI-digested lambda DNA [8] was then added to the mixture and reactions were incubated at room temperature for 30 minutes. Samples were analyzed via Western blotting using standard conditions.

#### GST pull-down assays

GST pull-down assays were performed exactly as described [11].

## Supporting information

S1 Fig Complete blots for Figs 2C and 6C. (TIF)

S2 Fig Replicate experiment for the one shown in Fig 3C. (TIF)

S3 Fig Uncropped gel images. (PDF)

S1 Raw images (PDF)

## References

[1] Paull TT. Mechanisms of ATM Activation. Annu Rev Biochem. 2015;84:711–38. doi: 10.1146/annurev-biochem-060614-034335 25580527

[2] Saldivar JC, Cortez D, Cimprich KA. The essential kinase ATR: ensuring faithful duplication of a challenging genome. Nat Rev Mol Cell Biol. 2017 Oct;18(10):622–636. doi: 10.1038/nrm.2017.67 28811666 PMC5796526

[3] Waterman DP, Haber JE, Smolka MB. Checkpoint Responses to DNA Double-Strand Breaks. Annu Rev Biochem. 2020 Jun 20;89:103–133. doi: 10.1146/annurev-biochem-011520-104722 32176524 PMC7311309

[4] Williams RM, Zhang X. Roles of ATM and ATR in DNA double strand breaks and replication stress. Prog Biophys Mol Biol. 2021 May;161:27–38. doi: 10.1016/j.pbiomolbio.2020.11.005 33259832

[5] Williams RM, Yates LA, Zhang X. Structures and regulations of ATM and ATR, master kinases in genome integrity. Curr Opin Struct Biol. 2020 Apr;61:98–105. doi: 10.1016/j.sbi.2019.12.010 31924595

[6] Lee JH, Paull TT. Cellular functions of the protein kinase ATM and their relevance to human disease. Nat Rev Mol Cell Biol. 2021 Dec;22(12):796–814. doi: 10.1038/s41580-021-00394-2 34429537

[7] Ma M, Rodriguez A, Sugimoto K. Activation of ATR-related protein kinase upon DNA damage recognition. Curr Genet. 2020 Apr;66(2):327–333. doi: 10.1007/s00294-019-01039-w 31624858 PMC7073305

[8] Montales K, Kim A, Ruis K, Michael WM. Structure-function analysis of TOPBP1’s role in ATR signaling using the DSB-mediated ATR activation in Xenopus egg extracts (DMAX) system. Sci Rep. 2021 Jan 11;11(1):467. doi: 10.1038/s41598-020-80626-1 33432091 PMC7801695

[9] Ruis K, Huynh O, Montales K, Barr NA, Michael WM. Delineation of a minimal topoisomerase II binding protein 1 (TOPBP1) for regulated activation of ATR at DNA double-strand breaks. J Biol Chem. 2022 Apr 28:101992. doi: 10.1016/j.jbc.2022.101992 35490781 PMC9257406

[10] Wardlaw CP, Carr AM, Oliver AW. TopBP1: A BRCT-scaffold protein functioning in multiple cellular pathways. DNA Repair (Amst). 2014 Oct;22:165–74. doi: 10.1016/j.dnarep.2014.06.004 25087188

[11] Kim A, Montales K, Ruis K, Senebandith H, Gasparyan H, Cowan Q, Michael WM. Biochemical analysis of TOPBP1 oligomerization. DNA Repair (Amst). 2020 Dec;96:102973. doi: 10.1016/j.dnarep.2020.102973 32987353 PMC7670859

[12] Thada V, Cortez D. ATR activation is regulated by dimerization of ATR activating proteins. J Biol Chem. 2021 Jan-Jun;296:100455. doi: 10.1016/j.jbc.2021.100455 33636182 PMC7994790

[13] Cescutti R, Negrini S, Kohzaki M, Halazonetis TD. TopBP1 functions with 53BP1 in the G1 DNA damage checkpoint. EMBO J. 2010 Nov 3;29(21):3723–32. doi: 10.1038/emboj.2010.238 20871591 PMC2982761

[14] Duursma AM, Driscoll R, Elias JE, Cimprich KA. A role for the MRN complex in ATR activation via TOPBP1 recruitment. Mol Cell. 2013 Apr 11;50(1):116–22. doi: 10.1016/j.molcel.2013.03.006 23582259 PMC3669687

[15] Shiotani B, Nguyen HD, Håkansson P, Maréchal A, Tse A, Tahara H, et al. Two distinct modes of ATR activation orchestrated by Rad17 and Nbs1. Cell Rep. 2013 May 30;3(5):1651–62. doi: 10.1016/j.celrep.2013.04.018 23684611 PMC3680100

[16] Yoo HY, Kumagai A, Shevchenko A, Shevchenko A, Dunphy WG. The Mre11-Rad50-Nbs1 complex mediates activation of TopBP1 by ATM. Mol Biol Cell. 2009 May;20(9):2351–60. doi: 10.1091/mbc.e08-12-1190 19279141 PMC2675615

[17] Taylor EM, Cecillon SM, Bonis A, Chapman JR, Povirk LF, Lindsay HD. The Mre11/Rad50/Nbs1 complex functions in resection-based DNA end joining in Xenopus laevis. Nucleic Acids Res. 2010 Jan;38(2):441–54. doi: 10.1093/nar/gkp905 19892829 PMC2811014

[18] You Z, Shi LZ, Zhu Q, Wu P, Zhang YW, Basilio A, et al. CtIP links DNA double-strand break sensing to resection. Mol Cell. 2009 Dec 25;36(6):954–69. doi: 10.1016/j.molcel.2009.12.002 20064462 PMC2807415

[19] Williams RS, Dodson GE, Limbo O, Yamada Y, Williams JS, Guenther G, et al. Nbs1 flexibly tethers Ctp1 and Mre11-Rad50 to coordinate DNA double-strand break processing and repair. Cell. 2009 Oct 2;139(1):87–99. doi: 10.1016/j.cell.2009.07.033 19804755 PMC2762657

[20] Jaz Jazayeri A, Falck J, Lukas C, Bartek J, Smith GC, Lukas J, et al. ATM- and cell cycle-dependent regulation of ATR in response to DNA double-strand breaks. Nat Cell Biol. 2006 Jan;8(1):37–45. doi: 10.1038/ncb1337 16327781

[21] Myers JS, Cortez D. Rapid activation of ATR by ionizing radiation requires ATM and Mre11. J Biol Chem. 2006 Apr 7;281(14):9346–50. doi: 10.1074/jbc.M513265200 16431910 PMC1821075

[22] Yoo HY, Kumagai A, Shevchenko A, Shevchenko A, Dunphy WG. Ataxia-telangiectasia mutated (ATM)-dependent activation of ATR occurs through phosphorylation of TopBP1 by ATM. J Biol Chem. 2007 Jun 15;282(24):17501–6. doi: 10.1074/jbc.M701770200 17446169

[23] Bakkenist CJ, Kastan MB. DNA damage activates ATM through intermolecular autophosphorylation and dimer dissociation. Nature. 2003 Jan 30;421(6922):499–506. doi: 10.1038/nature01368 12556884

[24] Dupré A, Boyer-Chatenet L, Sattler RM, Modi AP, Lee JH, Nicolette ML, et al. A forward chemical genetic screen reveals an inhibitor of the Mre11-Rad50-Nbs1 complex. Nat Chem Biol. 2008 Feb;4(2):119–25. doi: 10.1038/nchembio.63 18176557 PMC3065498

[25] Kumagai A, Dunphy WG. Claspin, a novel protein required for the activation of Chk1 during a DNA replication checkpoint response in Xenopus egg extracts. Mol Cell. 2000 Oct;6(4):839–49. doi: 10.1016/s1097-2765(05)00092-4 11090622

[26] Delacroix S, Wagner JM, Kobayashi M, Yamamoto K, Karnitz LM. The Rad9-Hus1-Rad1 (9-1-1) clamp activates checkpoint signaling via TopBP1. Genes Dev. 2007 Jun 15;21(12):1472–7. doi: 10.1101/gad.1547007 17575048 PMC1891424

[27] Lee J, Kumagai A, Dunphy WG. The Rad9-Hus1-Rad1 checkpoint clamp regulates interaction of TopBP1 with ATR. J Biol Chem. 2007 Sep 21;282(38):28036–44. doi: 10.1074/jbc.M704635200 17636252

[28] Ramírez-Lugo JS, Yoo HY, Yoon SJ, Dunphy WG. CtIP interacts with TopBP1 and Nbs1 in the response to double-stranded DNA breaks (DSBs) in Xenopus egg extracts. Cell Cycle. 2011 Feb 1;10(3):469–80. doi: 10.4161/cc.10.3.14711 21263215 PMC3115019

[29] Bigot N, Day M, Baldock RA, Watts FZ, Oliver AW, Pearl LH. Phosphorylation-mediated interactions with TOPBP1 couple 53BP1 and 9-1-1 to control the G1 DNA damage checkpoint. Elife. 2019 May 28;8:e44353. doi: 10.7554/eLife.44353 31135337 PMC6561707

[30] Blackford AN, Nieminuszczy J, Schwab RA, Galanty Y, Jackson SP, Niedzwiedz W. TopBP1 interacts with BLM to maintain genome stability but is dispensable for preventing BLM degradation. Mol Cell. 2015 Mar 19;57(6):1133–1141. doi: 10.1016/j.molcel.2015.02.012 25794620 PMC4374139

[31] Choi SH, Yoo HY. Mdc1 modulates the interaction between TopBP1 and the MRN complex during DNA damage checkpoint responses. Biochem Biophys Res Commun. 2016 Oct 7;479(1):5–11. doi: 10.1016/j.bbrc.2016.08.158 27590578

[32] Leimbacher PA, Jones SE, Shorrocks AK, de Marco Zompit M, Day M, Blaauwendraad J, et al. MDC1 Interacts with TOPBP1 to Maintain Chromosomal Stability during Mitosis. Mol Cell. 2019 May 2;74(3):571–583.e8. doi: 10.1016/j.molcel.2019.02.014 30898438 PMC6509287

[33] Day M, Rappas M, Ptasinska K, Boos D, Oliver AW, Pearl LH. BRCT domains of the DNA damage checkpoint proteins TOPBP1/Rad4 display distinct specificities for phosphopeptide ligands. Elife. 2018 Oct 8;7:e39979. doi: 10.7554/eLife.39979 30295604 PMC6175577

[34] Wühr M, Freeman RM Jr, Presler M, Horb ME, Peshkin L, Gygi S, et al. Deep proteomics of the Xenopus laevis egg using an mRNA-derived reference database. Curr Biol. 2014 Jul 7;24(13):1467–1475. doi: 10.1016/j.cub.2014.05.044 24954049 PMC4090281

[35] Blaikley EJ, Tinline-Purvis H, Kasparek TR, Marguerat S, Sarkar S, Hulme L, et al. The DNA damage checkpoint pathway promotes extensive resection and nucleotide synthesis to facilitate homologous recombination repair and genome stability in fission yeast. Nucleic Acids Res. 2014 May;42(9):5644–56. doi: 10.1093/nar/gku190 24623809 PMC4027169

[36] Gobbini E, Casari E, Colombo CV, Bonetti D, Longhese MP. The 9-1-1 Complex Controls Mre11 Nuclease and Checkpoint Activation during Short-Range Resection of DNA Double-Strand Breaks. Cell Rep. 2020 Oct 20;33(3):108287. doi: 10.1016/j.celrep.2020.108287 33086066

[37] Takeishi Y, Ohashi E, Ogawa K, Masai H, Obuse C, Tsurimoto T. Casein kinase 2-dependent phosphorylation of human Rad9 mediates the interaction between human Rad9-Hus1-Rad1 complex and TopBP1. Genes Cells. 2010 Jun;15(7):761–71. doi: 10.1111/j.1365-2443.2010.01418.x 20545769

[38] Zhao J, Tian S, Guo Q, Bao K, Yu G, Wang X, et al. A PARylation-phosphorylation cascade promotes TOPBP1 loading and RPA-RAD51 exchange in homologous recombination. Mol Cell. 2022 May 13:S1097-2765(22)00394-X. doi: 10.1016/j.molcel.2022.04.031 35597237

[39] Lee J, Dunphy WG. The Mre11-Rad50-Nbs1 (MRN) complex has a specific role in the activation of Chk1 in response to stalled replication forks. Mol Biol Cell. 2013 May;24(9):1343–53. doi: 10.1091/mbc.E13-01-0025 23468519 PMC3639046

[40] Shiotani B, Zou L. Single-stranded DNA orchestrates an ATM-to-ATR switch at DNA breaks. Mol Cell. 2009 Mar 13;33(5):547–58. doi: 10.1016/j.molcel.2009.01.024 19285939 PMC2675165

[41] Liao S, Guay C, Toczylowski T, Yan H. Analysis of MRE11’s function in the 5’—>3’ processing of DNA double-strand breaks. Nucleic Acids Res. 2012 May;40(10):4496–506. doi: 10.1093/nar/gks044 22319209 PMC3378884

[42] Frattini C, Promonet A, Alghoul E, Vidal-Eychenie S, Lamarque M, Blanchard MP, et al. TopBP1 assembles nuclear condensates to switch on ATR signaling. Mol Cell. 2021 Mar 18;81(6):1231–1245.e8. doi: 10.1016/j.molcel.2020.12.049 33503405

[43] Van Hatten RA, Tutter AV, Holway AH, Khederian AM, Walter JC, Michael WM. The Xenopus Xmus101 protein is required for the recruitment of Cdc45 to origins of DNA replication. J Cell Biol. 2002 Nov 25;159(4):541–7. doi: 10.1083/jcb.200207090 12438414 PMC2173091

[44] Smythe C, Newport JW. Systems for the study of nuclear assembly, DNA replication, and nuclear breakdown in Xenopus laevis egg extracts. Methods Cell Biol. 1991;35:449–68. doi: 10.1016/s0091-679x(08)60583-x 1664032

