# Supplementary figures and images for: MRN-dependent and independent pathways for recruitment of TOPBP1 to DNA double-strand breaks
Source: PLoS One. 2022 Aug 2;17(8):e0271905. doi: 10.1371/journal.pone.0271905 (PMC9345342; doi:10.1371/journal.pone.0271905)

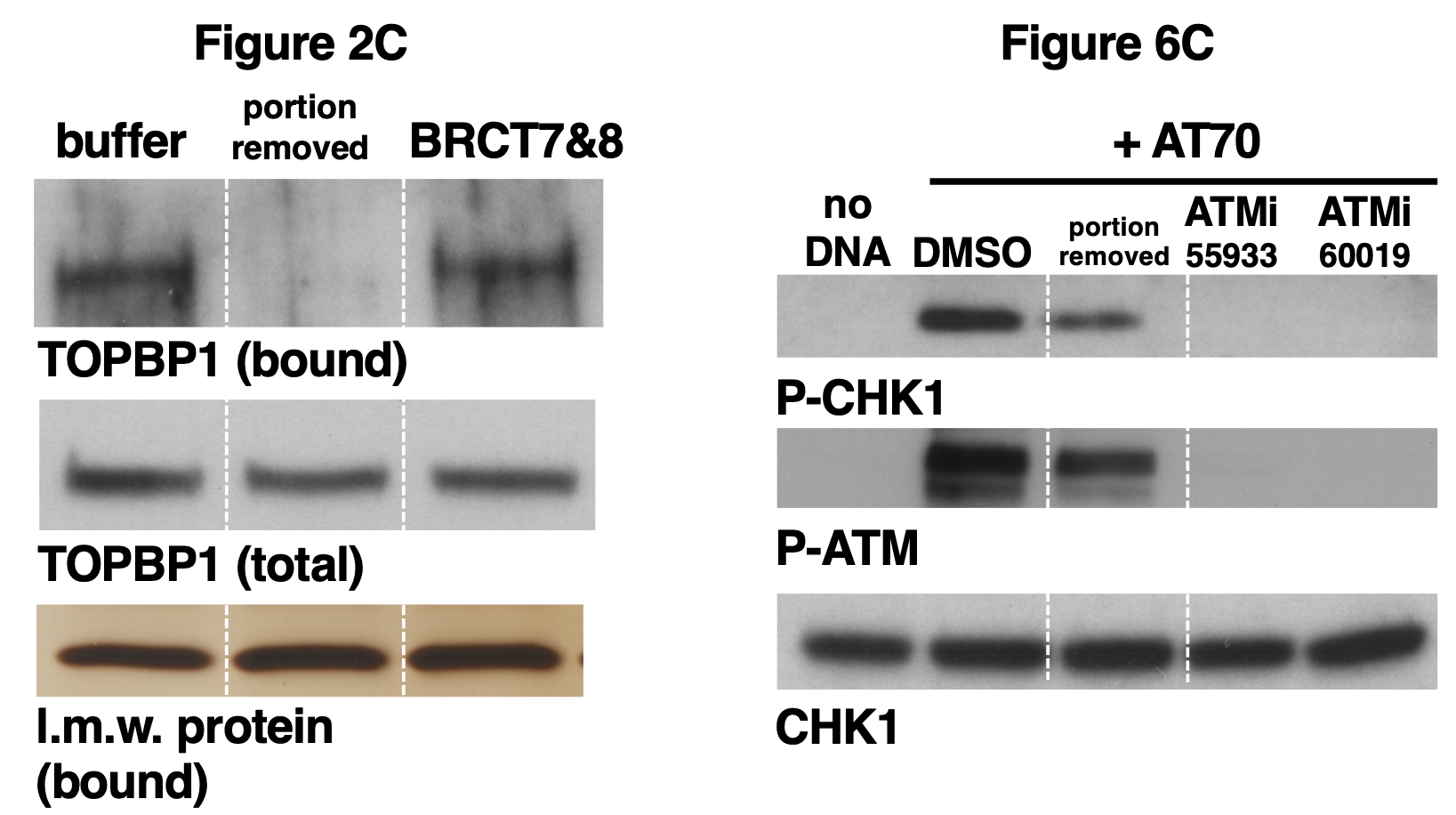

Supplement: S1 Fig — (TIF) [file pone.0271905.s001.tif]

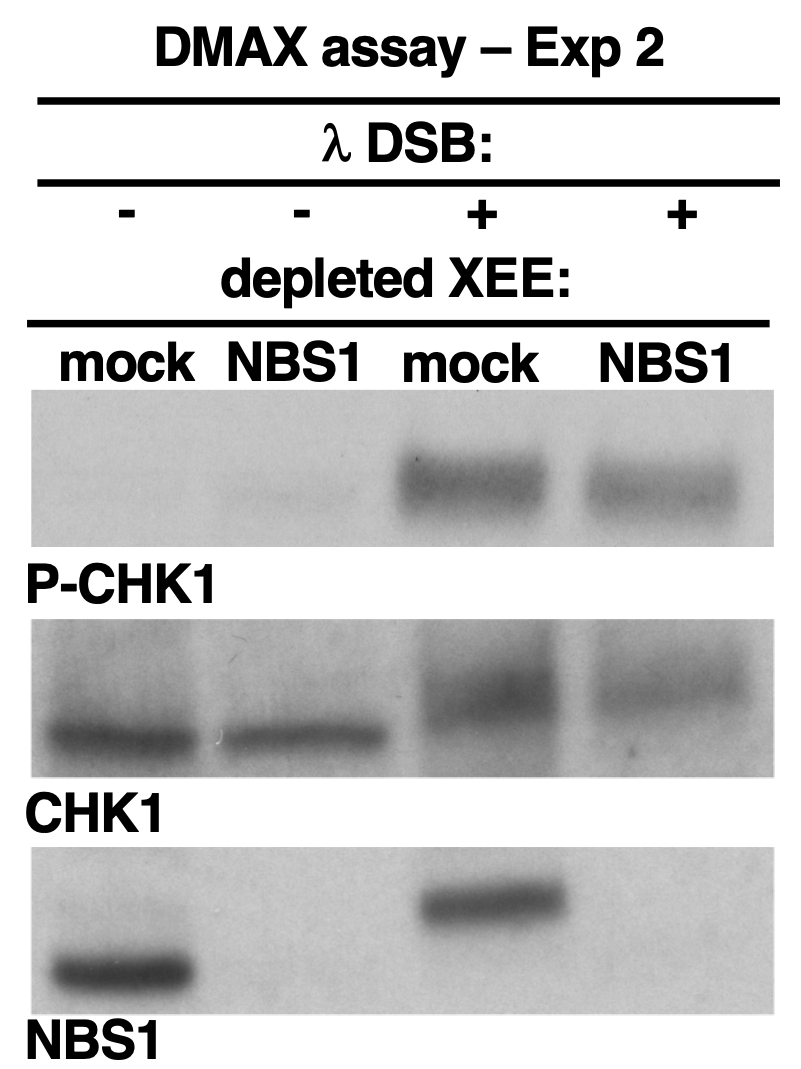

Supplement: S2 Fig — (TIF) [file pone.0271905.s002.tif]
