# Supplementary material for: MRN-dependent and independent pathways for recruitment of TOPBP1 to DNA double-strand breaks
Source: PLoS One. 2022 Aug 2;17(8):e0271905. doi: 10.1371/journal.pone.0271905 (PMC9345342; doi:10.1371/journal.pone.0271905)
Supplement: S3 Fig — (PDF) [file pone.0271905.s003.pdf]

Fig 2B

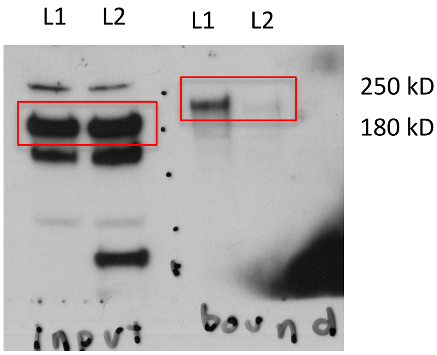

Left pair
Lane 1: total extract containing buffer probed for TOPBP1.
Lane 2: total extract containing BRCT0-2 probed for TOPBP1.
Right pair
Lane 1: DSB bound fraction containing buffer probed for TOPBP1.
Lane 2: DSB bound fraction containing BRCT0-2 probed for TOPBP1.

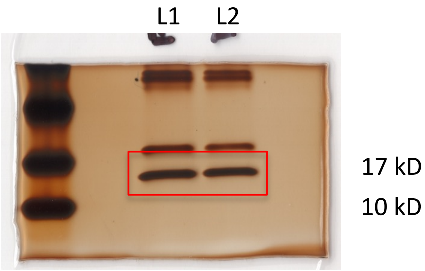

Lane 1: DSB bound fraction containing buffer stained with silver.
Lane 2: DSB bound fraction containing BRCT0-2 stained with silver.

Fig 2D continued

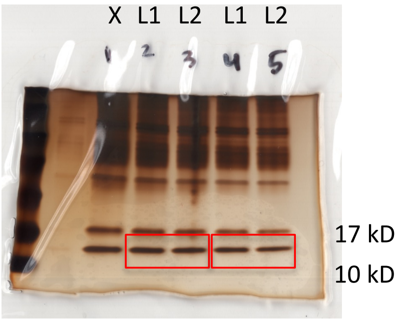

Left pair
Lane 1: total extract containing buffer, T0/T30, stained with silver.
Lane 2: total extract containing BRCT0-2, T0/T30 stained with silver.
Right pair
Lane 1: DSB bound fraction containing buffer, T30/T60, stained with silver.
Lane 2: DSB bound fraction containing BRCT0-2, T30/T60, stained with silver.

Fig 2C

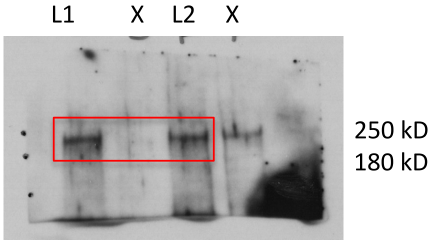

Lane 1: DSB bound fraction containing buffer probed for TOPBP1.
Lane 2: DSB bound fraction containing BRCT7&8 probed for TOPBP1.

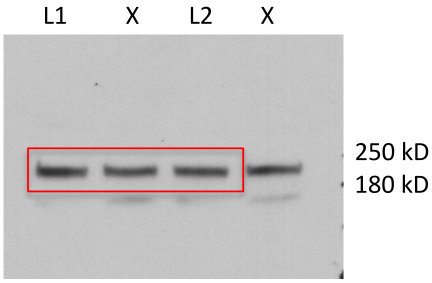

Lane 1: total extract containing buffer probed for TOPBP1.
Lane 2: total extract containing BRCT7&8 probed for TOPBP1.

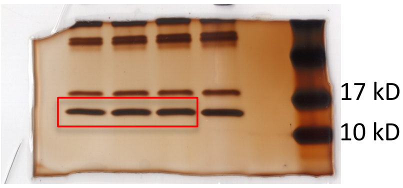

Lane 1: DSB bound fraction containing buffer stained with silver.
Lane 2: DSB bound fraction containing BRCT07&8 stained with silver.

Fig 2D

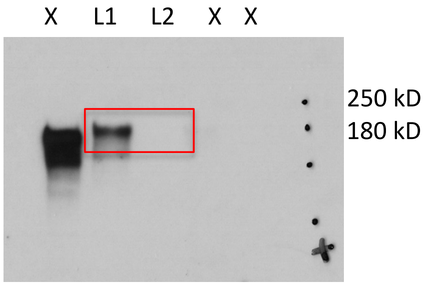

Lane 1: DSB bound fraction containing buffer, T0/T30, probed for TOPBP1.
Lane 2: DSB bound fraction containing BRCT0-2, T0/T30, probed for TOPBP1.

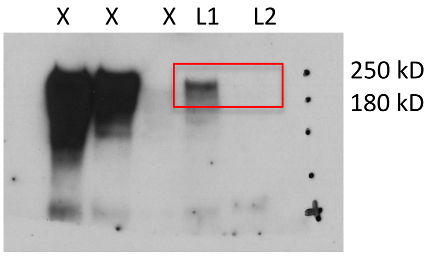

Lane 1: DSB bound fraction containing buffer, T30/T60, probed for TOPBP1.
Lane 2: DSB bound fraction containing BRCT0-2, T30/T60, probed for TOPBP1.

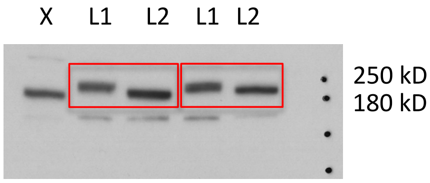

Left pair
Lane 1: total extract containing buffer, T0/T30, probed for TOPBP1.
Lane 2: total extract containing BRCT0-2, T0/T30 probed for TOPBP1.
Right pair
Lane 1: DSB bound fraction containing buffer, T30/T60, probed for TOPBP1.
Lane 2: DSB bound fraction containing BRCT0-2, T30/T60, probed for TOPBP1.

Fig 2E

L1 L2 L3 L4 L5

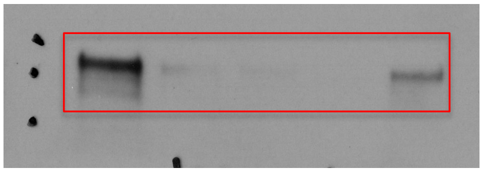

250 kD
180 kD

Lane 1 DSB bound fraction from sample containing buffer, T30.
Lane 2 DSB bound fraction from sample containing BRCT0-2, T40.
Lane 3 DSB bound fraction from sample containing BRCT0-2, T50.
Lane 4 DSB bound fraction from sample containing BRCT0-2, T60.
Lane 5 DSB bound fraction from sample containing buffer, T30/T60
All probed for TOPBP1.

L1 L2 L3 L4 L5

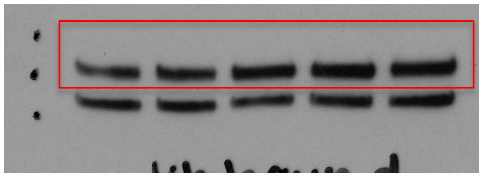

250 kD
180 kD

Lane 1 Total extract from sample containing buffer, T30.
Lane 2 Total extract from sample containing BRCT0-2, T40.
Lane 3 Total extract from sample containing BRCT0-2, T50.
Lane 4 total extract from sample containing BRCT0-2, T60.
Lane 5 total extract from sample containing buffer, T30/T60
All probed for TOPBP1.

L1 L2 L3 L4 L5

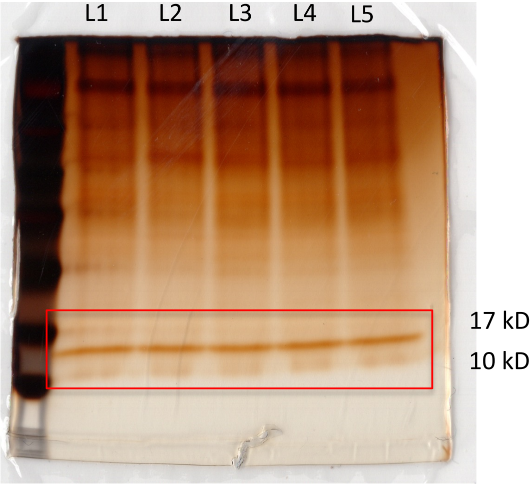

17 kD
10 kD

Lane 1 DSB bound fraction from sample containing buffer, T30.
Lane 2 DSB bound fraction from sample containing BRCT0-2, T40.
Lane 3 DSB bound fraction from sample containing BRCT0-2, T50.
Lane 4 DSB bound fraction from sample containing BRCT0-2, T60.
Lane 5 DSB bound fraction from sample containing buffer, T30/T60
Stained with silver.

Fig 3A

L1 L2

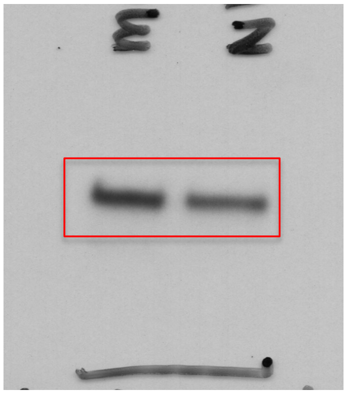

250 kD
180 kD

Lane 1: total extract from mock-depleted sample.
Lane 2: total extract from NBS1-depleted sample.
Probed for TOPBP1.

L1 L2

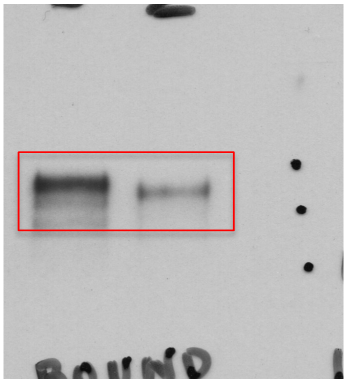

250 kD
180 kD

Lane 1: DSB bound sample from mock-depleted sample.
Lane 2: DSB bound sample from NBS1-depleted sample.
Probed for TOPBP1.

L1 L2

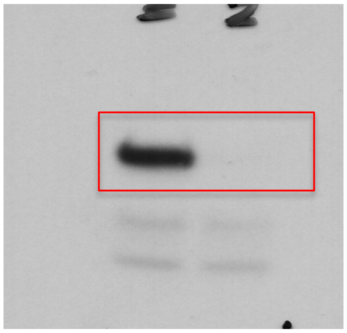

95 kD
72 kD

Lane 1: total extract from mock-depleted sample.
Lane 2: total extract from NBS1-depleted sample.
Probed for MRE11.

L1 L2

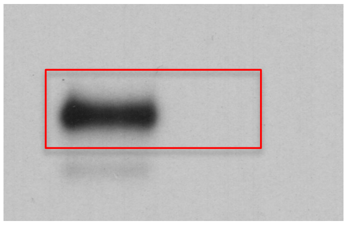

95 kD
72 kD

Lane 1: DSB bound sample from mock-depleted sample.
Lane 2: DSB bound sample from NBS1-depleted sample.
Probed for MRE11.

L1 L2 L1 L2

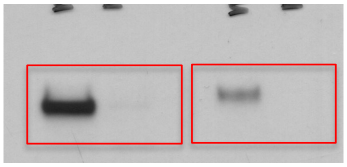

130 kD
95 kD

Left side
Lane 1: total extract from mock-depleted sample.
Lane 2: total extract from NBS1-depleted sample.
Probed for NBS1.
Right side
Lane 1: DSB bound sample from mock-depleted sample.
Lane 2: DSB bound sample from NBS1-depleted sample.
All probed for NBS1.

L1 L2

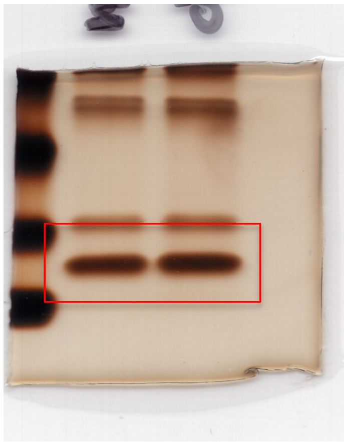

17 kD
10 kD

Lane 1: DSB bound sample from mock-depleted sample.
Lane 2: DSB bound sample from NBS1-depleted sample.
Stained with silver.

Fig 3B

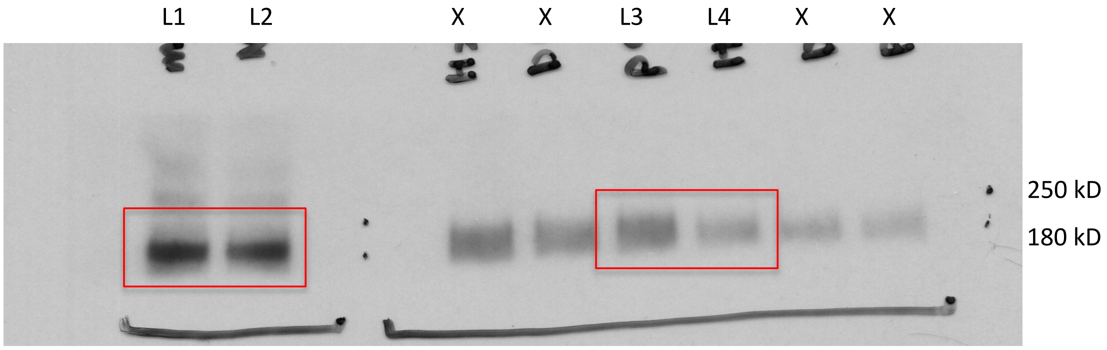

Lane 1 Mock depleted total extract, Lane 2 NBS1-depleted total extract, Lane 3 DSB bound sample from mock depleted extract, Lane 4 DSB bound sample from NBS1 depleted extract. Probed for TOPBP1.

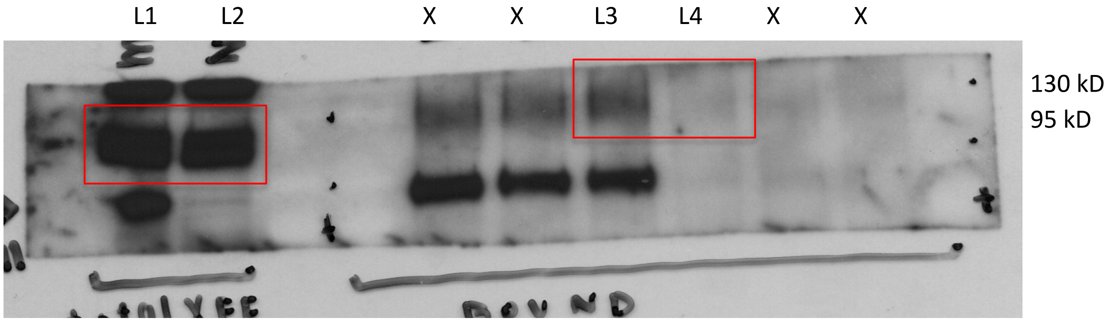

Lane 1 Mock depleted total extract, Lane 2 NBS1-depleted total extract, Lane 3 DSB bound sample from mock depleted extract, Lane 4 DSB bound sample from NBS1 depleted extract. Probed for CtIP.

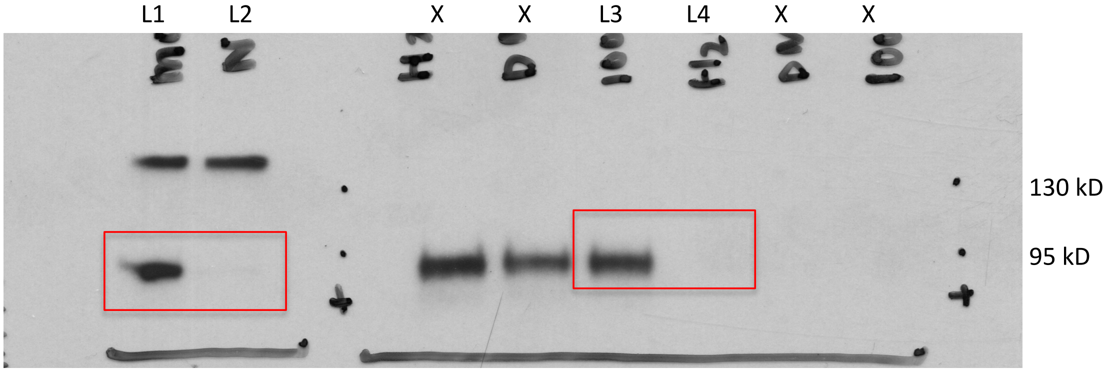

Lane 1 Mock depleted total extract, Lane 2 NBS1-depleted total extract, Lane 3 DSB bound sample from mock depleted extract, Lane 4 DSB bound sample from NBS1 depleted extract. Probed for NBS1.

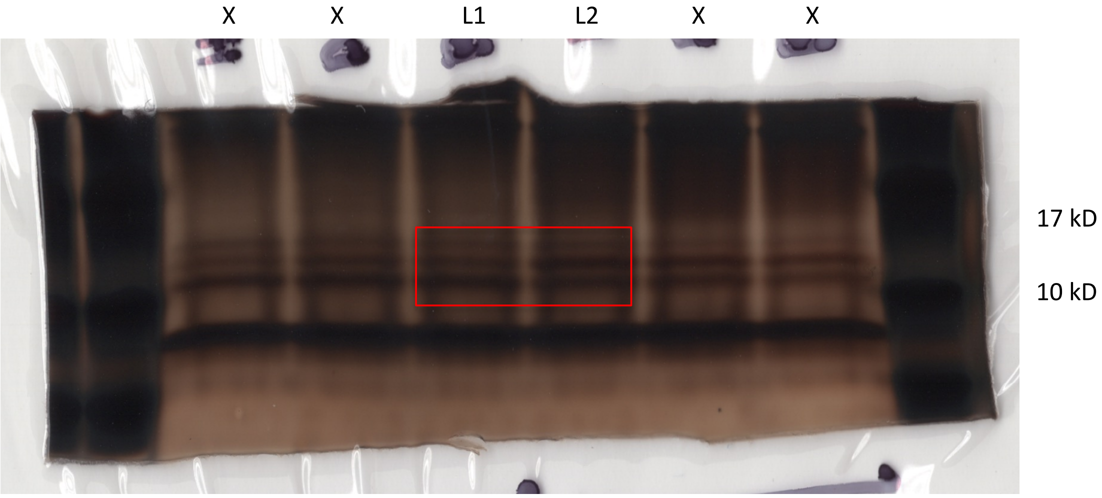

Lane 1 DSB bound sample from mock depleted extract, Lane 2 DSB bound sample from NBS1 depleted extract. Stained with silver.

Fig 3C

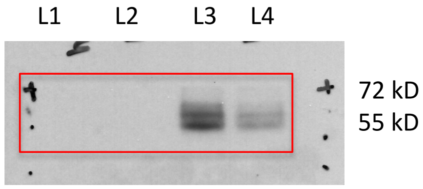

Lane 1 Total mock extract, no DNA.
Lane 2 Total NBS1 depleted extract, no DNA.
Lane 3 Total mock extract, plus DNA.
Lane 4 Total NBS depleted extract, plus DNA.
Probed for P- CHK1.

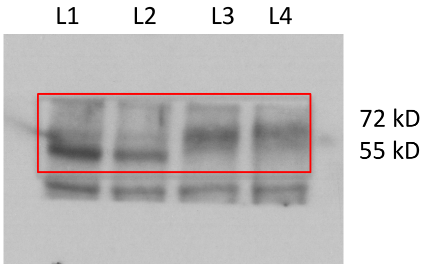

Lane 1 Total mock extract, no DNA.
Lane 2 Total NBS1 depleted extract, no DNA.
Lane 3 Total mock extract, plus DNA.
Lane 4 Total NBS depleted extract, plus DNA.
Probed for CHK1.

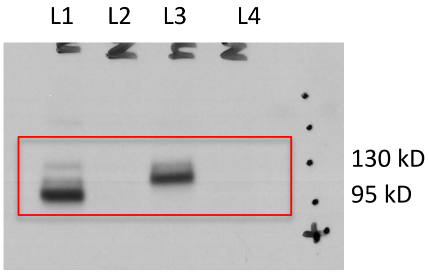

Lane 1 Total mock extract, no DNA.
Lane 2 Total NBS1 depleted extract, no DNA.
Lane 3 Total mock extract, plus DNA.
Lane 4 Total NBS depleted extract, plus DNA.
Probed for NBS1.

Fig S2

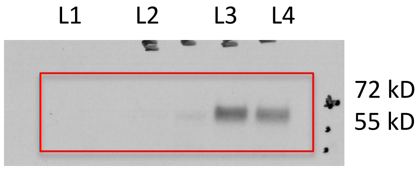

Lane 1 Total mock extract, no DNA.
Lane 2 Total NBS1 depleted extract, no DNA.
Lane 3 Total mock extract, plus DNA.
Lane 4 Total NBS depleted extract, plus DNA.
Probed for P- CHK1.

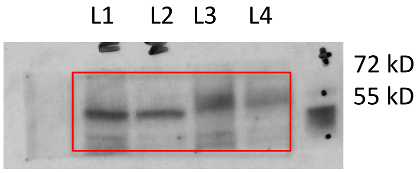

Lane 1 Total mock extract, no DNA.
Lane 2 Total NBS1 depleted extract, no DNA.
Lane 3 Total mock extract, plus DNA.
Lane 4 Total NBS depleted extract, plus DNA.
Probed for CHK1.

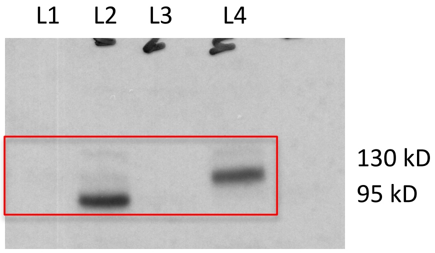

Lane 1 Total mock extract, no DNA.
Lane 2 Total NBS1 depleted extract, no DNA.
Lane 3 Total mock extract, plus DNA.
Lane 4 Total NBS depleted extract, plus DNA.
Probed for NBS1.

Fig 3D

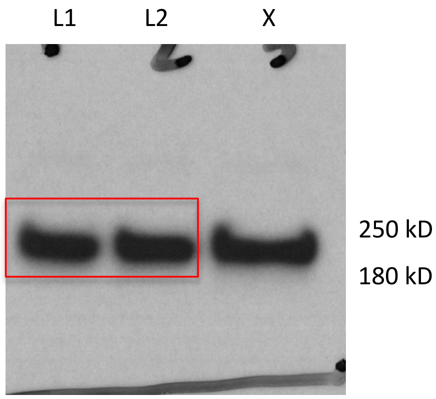

Lane 1 Total mock depleted extract.
Lane 2 Total CtIP depleted extract.
Probed for TOPBP1.

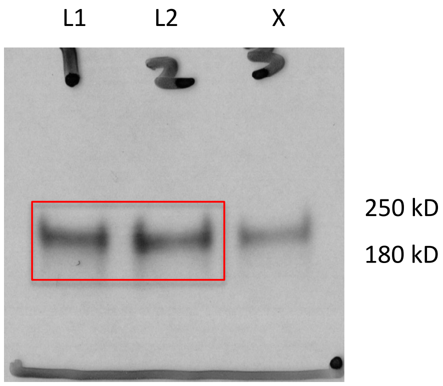

Lane 1 DSB bound from mock depleted extract.
Lane 2 DSB bound from CtIP depleted extract.
Probed for TOPBP1.

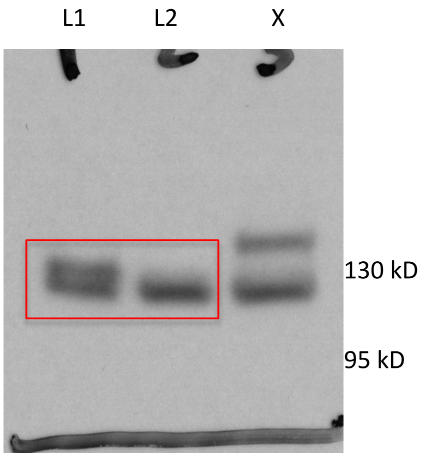

Lane 1 Total mock depleted extract.
Lane 2 Total CtIP depleted extract.
Probed for CtIP.

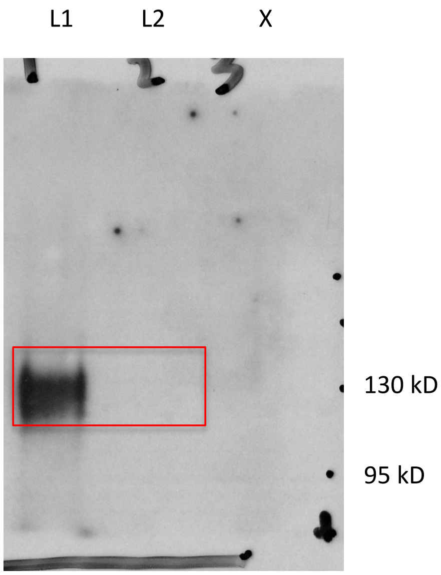

Lane 1 DSB bound from mock depleted extract.
Lane 2 DSB bound from CtIP depleted extract.
Probed for CtIP.

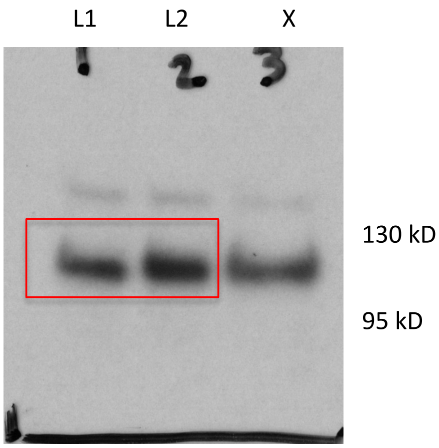

Lane 1 Total mock depleted extract.
Lane 2 Total CtIP depleted extract.
Probed for NBS1.

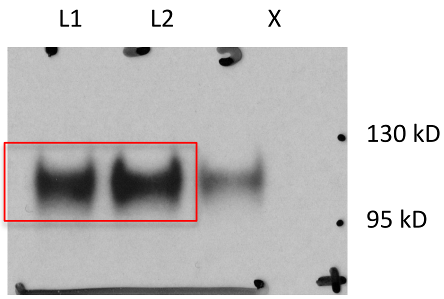

Lane 1 DSB bound from mock depleted extract.
Lane 2 DSB bound from CtIP depleted extract.
Probed for NBS1.

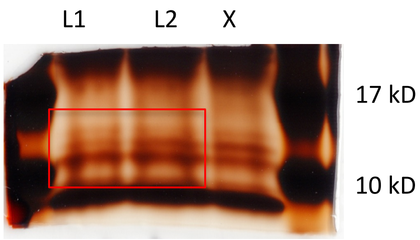

Lane 1 DSB bound from mock depleted extract.
Lane 2 DSB bound from CtIP depleted extract.
Stained with silver.

Fig 4A

L1 L2 L3

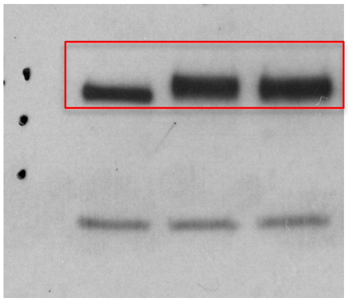

250 kD
180 kD

Lane 1 Total extract that had empty beads.
Lane 2 Total extract with DNA beads and DMSO.
Lane 3 Total extract with DNA beads and ATMi.
Probed for TOPBP1.

L1 L2 L3 X

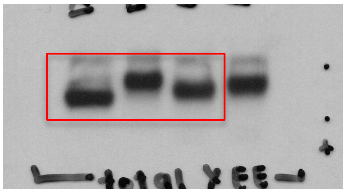

130 kD
95 kD

Lane 1 Total extract that had empty beads.
Lane 2 Total extract with DNA beads and DMSO.
Lane 3 Total extract with DNA beads and ATMi.
Probed for NBS1.

L1 L2 L3 X

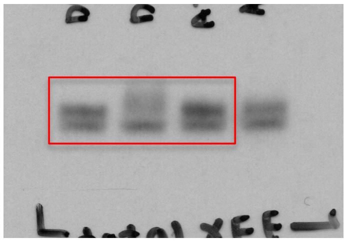

130 kD
95 kD

Lane 1 Total extract that had empty beads.
Lane 2 Total extract with DNA beads and DMSO.
Lane 3 Total extract with DNA beads and ATMi.
Probed for CtIP.

L1 L2 L3 X

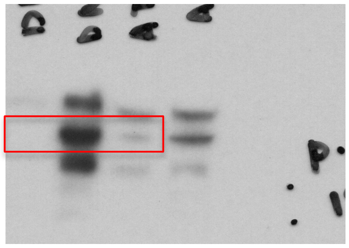

250 kD

Lane 1 Total extract that had empty beads.
Lane 2 Total extract with DNA beads and DMSO.
Lane 3 Total extract with DNA beads and ATMi.
Probed for P-ATM.

X L1 L2 L3 X

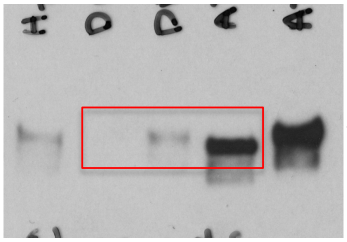

250 kD
180 kD

Lane 1 DSB-bound fractions from extract with empty beads.
Lane 2 DSB-bound fraction from extract with DNA beads and DMSO.
Lane 3 DSB-bound fraction from extract with DNA beads and ATMi.
Probed for TOPBP1.

L1 L2 L3 X

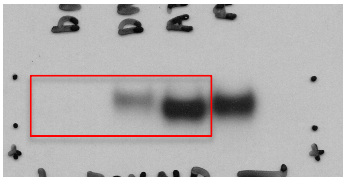

130 kD
95 kD

Lane 1 DSB-bound fractions from extract with empty beads.
Lane 2 DSB-bound fraction from extract with DNA beads and DMSO.
Lane 3 DSB-bound fraction from extract with DNA beads and ATMi.
Probed for NBS1.

L1 L2 L3 X

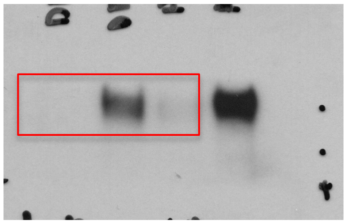

130 kD
95 kD

Lane 1 DSB-bound fractions from extract with empty beads.
Lane 2 DSB-bound fraction from extract with DNA beads and DMSO.
Lane 3 DSB-bound fraction from extract with DNA beads and ATMi.
Probed for CtIP.

L1 L2 L3 X

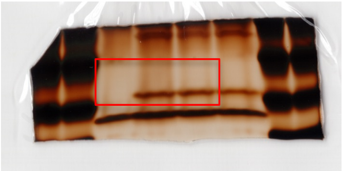

17 kD
10 kD

Lane 1 DSB-bound fractions from extract with empty beads.
Lane 2 DSB-bound fraction from extract with DNA beads and DMSO.
Lane 3 DSB-bound fraction from extract with DNA beads and ATMi.
Stained with silver.

Fig 4B

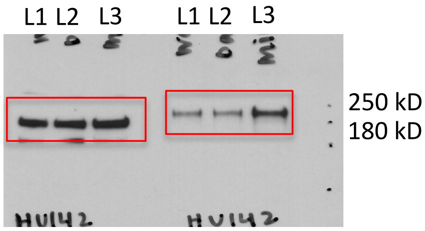

Left
Lane 1: total extract containing water.
Lane 2: total extract containing DMSO.
Total extract containing mirin.
Right
Lane 1: DSB bound fraction containing water.
Lane 2: DSB bound fraction containing DMSO
Lane 3: DSB bound fraction containing mirin.
Probed for TOPBP1.

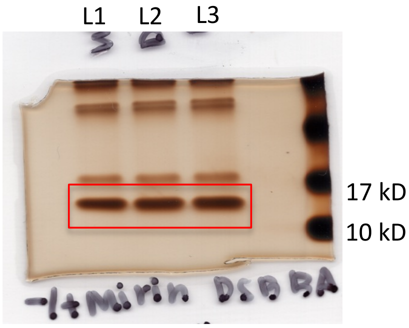

Lane 1: DSB bound fraction containing water.
Lane 2: DSB bound fraction containing DMSO
Lane 3: DSB bound fraction containing mirin.
Stained with silver.

Fig 5A

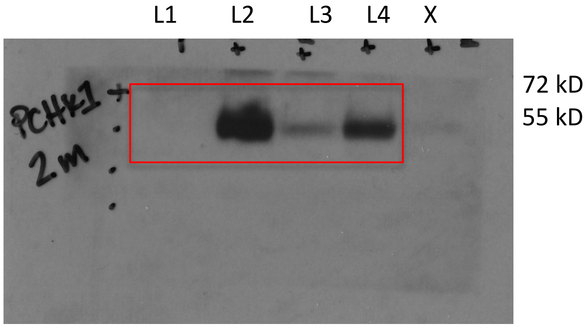

Lane 1 Total extract with nothing else.
Lane 2 Total extract with DMSO plus DNA.
Lane 3 Total extract with ATRi and DNA.
Lane 4 Total extract with ATMi plus DNA.
Probed for P-CHK1.

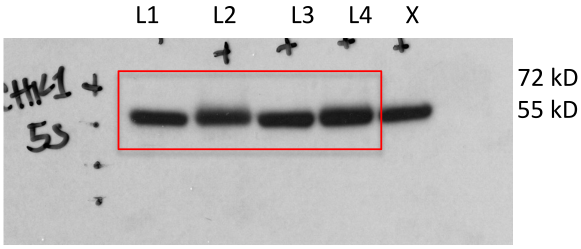

Lane 1 Total extract with nothing else.
Lane 2 Total extract with DMSO plus DNA.
Lane 3 Total extract with ATRi and DNA.
Lane 4 Total extract with ATMi plus DNA.
Probed for CHK1.

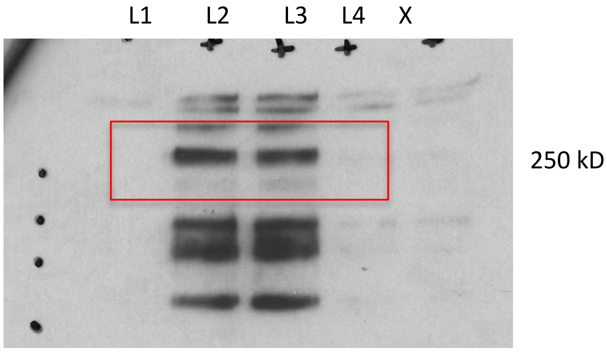

Lane 1 Total extract with nothing else.
Lane 2 Total extract with DMSO plus DNA.
Lane 3 Total extract with ATRi and DNA.
Lane 4 Total extract with ATMi plus DNA.
Probed for P-ATM.

Fig 5B

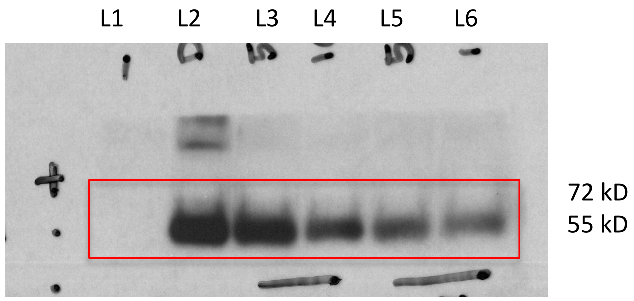

Lane 1 Total extract with nothing else.
Lane 2 Total extract with DMSO plus DNA.
Lane 3 Total extract with KU55933 (50uM) and DNA.
Lane 4 Total extract with KU55933 (100uM) and DNA.
Lane 3 Total extract with KU60019 (50uM) and DNA.
Lane 4 Total extract with KU60019 (100uM) and DNA.
Probed for P-CHK1.

Total extract probed for P-CHK1

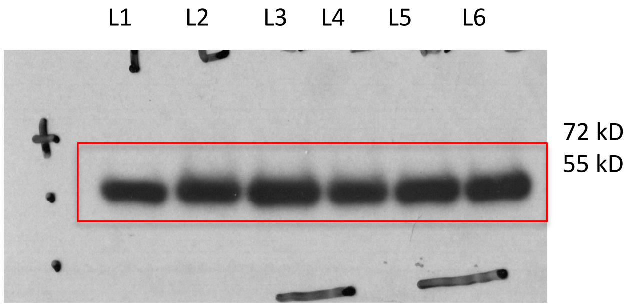

Lane 1 Total extract with nothing else.
Lane 2 Total extract with DMSO plus DNA.
Lane 3 Total extract with KU55933 (50uM) and DNA.
Lane 4 Total extract with KU55933 (100uM) and DNA.
Lane 3 Total extract with KU60019 (50uM) and DNA.
Lane 4 Total extract with KU60019 (100uM) and DNA.
Probed for CHK1.

Total extract probed for CHK1

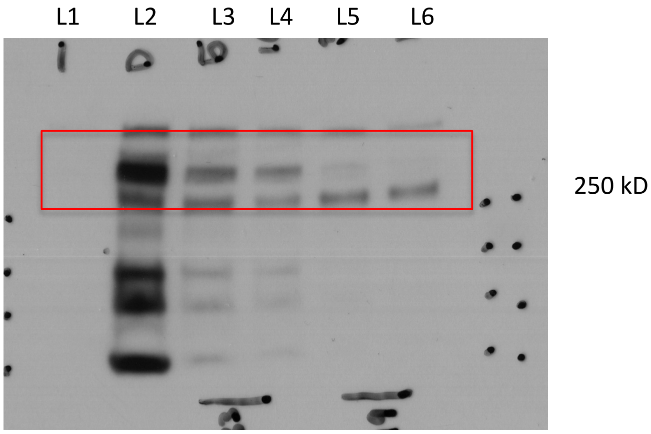

Lane 1 Total extract with nothing else.
Lane 2 Total extract with DMSO plus DNA.
Lane 3 Total extract with KU55933 (50uM) and DNA.
Lane 4 Total extract with KU55933 (100uM) and DNA.
Lane 3 Total extract with KU60019 (50uM) and DNA.
Lane 4 Total extract with KU60019 (100uM) and DNA.
Probed for P-ATM.

Total extract probed for P-ATM

Fig 6B

L1 L2 L3 L4

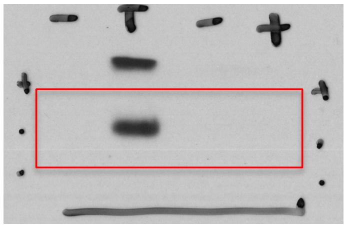

72 kD
55 kD

Lane 1 Total mock depleted extract.
Lane 2 Total mock depleted extract plus AT70.
Lane 3 Total NBS1 depleted extract.
Lane 4 Total NBS1 depleted extract plus AT70.
Probed for P-CHK1.

L1 L2 L3 L4

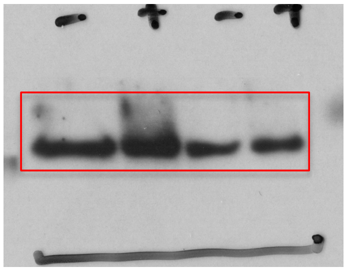

72 kD
55 kD

Lane 1 Total mock depleted extract.
Lane 2 Total mock depleted extract plus AT70.
Lane 3 Total NBS1 depleted extract.
Lane 4 Total NBS1 depleted extract plus AT70.
Probed for CHK1

L1 L2 L3 L4

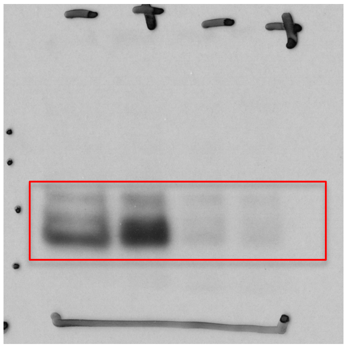

130 kD
95 kD

Lane 1 Total mock depleted extract.
Lane 2 Total mock depleted extract plus AT70.
Lane 3 Total NBS1 depleted extract.
Lane 4 Total NBS1 depleted extract plus AT70.
Probed for NBS1.

Fig 6C

L1 L2 X L3 L4

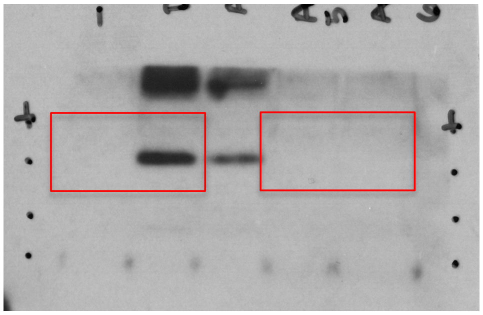

72 kD
55 kD

Lane 1 Total extract no DNA.
Lane 2 Total extract plus DMSO and DNA.
Lane 3 Total extract with ATMi 55933 and DNA.
Lane 4 Total extract with ATMi 60019 and DNA.
Probed for P-CHK1.

L1 L2 X L3 L4

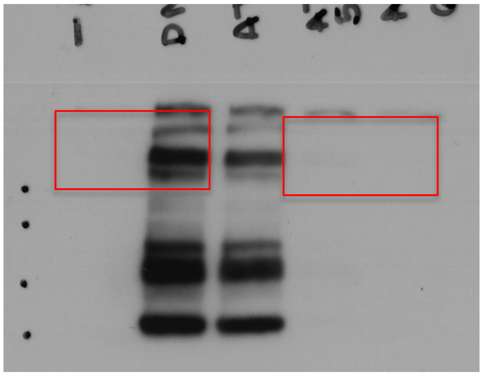

250 kD

Lane 1 Total extract no DNA.
Lane 2 Total extract plus DMSO and DNA.
Lane 3 Total extract with ATMi 55933 and DNA.
Lane 4 Total extract with ATMi 60019 and DNA.
Probed for P-ATM.

L1 L2 X L3 L4

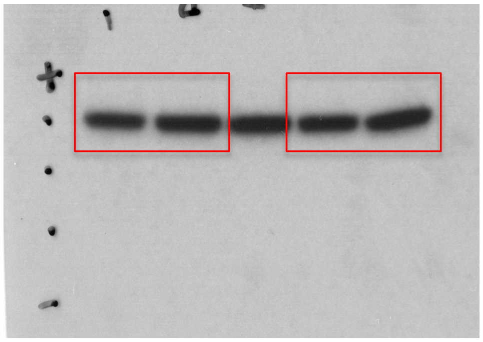

72 kD
55 kD

Lane 1 Total extract no DNA.
Lane 2 Total extract plus DMSO and DNA.
Lane 3 Total extract with ATMi 55933 and DNA.
Lane 4 Total extract with ATMi 60019 and DNA.
Probed for CHK1.

Fig 6D

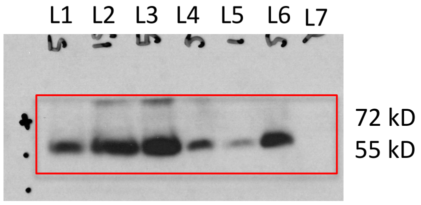

Lane 1 Total extract with dsDNA (50nM).
Lane 2 Total extract with dsDNA (150nM).
Lane 3 Total extract with dsDNA (500nM).
Lane 4 Total extract with AT70 (50nM).
Lane 5 Total extract with AT70 (150nM).
Lane 6 Total extract with AT70 (500nM).
Lane 7 Total extract no DNA.
Probed for P-CHK1.

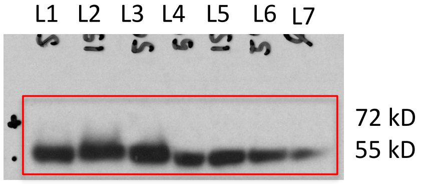

Lane 1 Total extract with dsDNA (50nM).
Lane 2 Total extract with dsDNA (150nM).
Lane 3 Total extract with dsDNA (500nM).
Lane 4 Total extract with AT70 (50nM).
Lane 5 Total extract with AT70 (150nM).
Lane 6 Total extract with AT70 (500nM).
Lane 7 Total extract no DNA.
Probed for CHK1.

Fig 7A

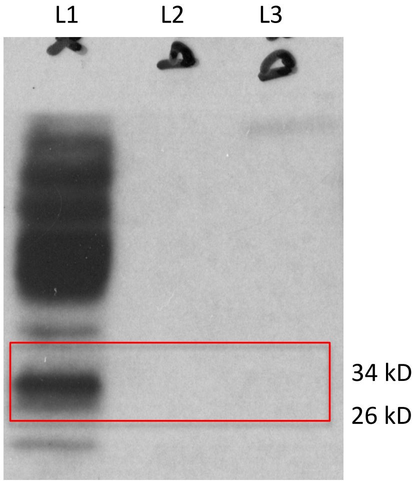

Lane 1 Total extract.
Lane 2 Empty bead fraction.
Lane 3 DSB bead fraction.
Probed for RAD1.

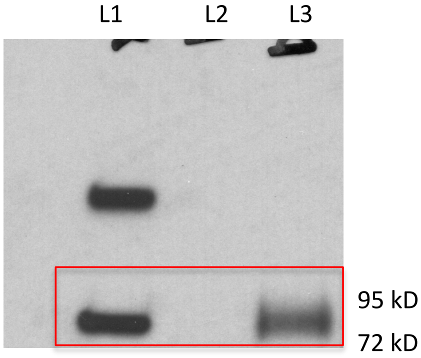

Lane 1 Total extract.
Lane 2 Empty bead fraction.
Lane 3 DSB bead fraction.
Probed for MRE11.

Fig 7B

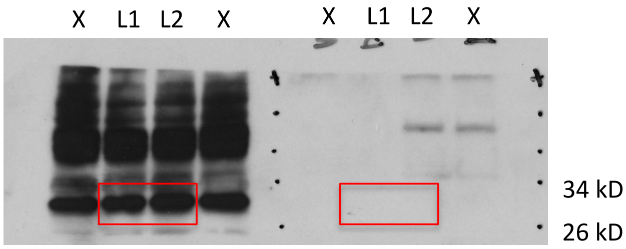

Lane 1 Total extract with empty beads.
Lane 2 Total extract with DSB beads.
Probed for RAD1.

Lane 1 Empty bead fraction.
Lane 2 DSB bead fraction..
Probed for RAD1.

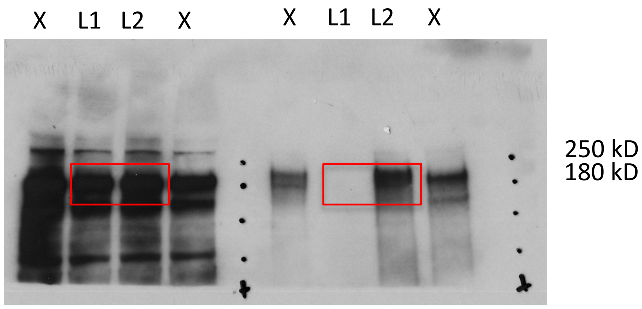

Lane 1 Total extract with empty beads.
Lane 2 Total extract with DSB beads.
Probed for TOPBP1.

Lane 1 Empty bead fraction.
Lane 2 DSB bead fraction..
Probed for TOPBP1.

Fig 7C

L1 L2 L3 L4

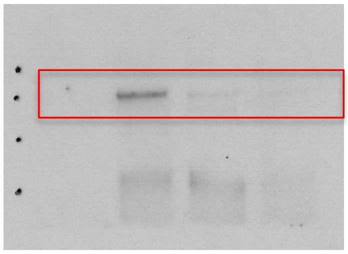

250 kD
180 kD

Lane 1 GST bound fraction.
Lane 2 GST-RAD9T bound fraction.
Lane 3 GST-RAD9T bound fraction plus CK2i 50 uM.
Lane 4 GST-RAD9T bound fraction plus CK2i 100 uM.
Probed for TOPBP1.

L1 L2 L3 L4

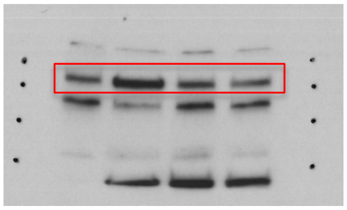

250 kD
180 kD

Lane 1 Total extract plus GST.
Lane 2 Total extract plus GST-RAD9T.
Lane 3 Total extract plus GST-RAD9T and CK2i 50 uM.
Lane 4 Total extract plus GST-RAD9T and CK2i 100 uM.
Probed for TOPBP1.

L1 L2 L3 L4

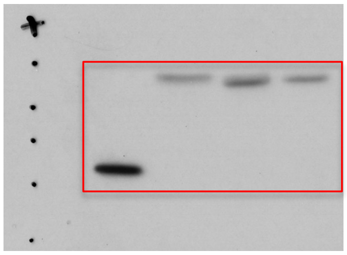

55 kD
34 kD

Lane 1 GST bound fraction.
Lane 2 GST-RAD9T bound fraction.
Lane 3 GST-RAD9T bound fraction plus CK2i 50 uM.
Lane 4 GST-RAD9T bound fraction plus CK2i 100 uM.
Probed for GST.

Fig 7D

L1 L2 L3

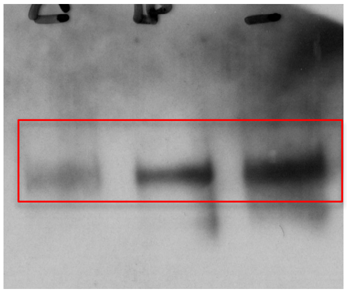

250 kD
180 kD

Lane 1 DSB bound fraction.
Lane 2 DSB bound fraction plus CK2i 50 uM.
Lane 4 DSB bound fraction plus CK2i 100 uM.
Probed for TOPBP1.

L1 L2 L3

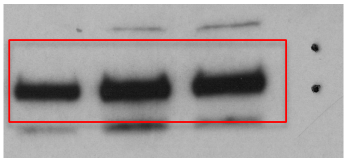

250 kD
180 kD

Lane 1 Total extract.
Lane 2 Total extract plus CK2i 50 uM.
Lane 4 Total extract plus CK2i 100 uM.
Probed for TOPBP1.

L1 L2 L3

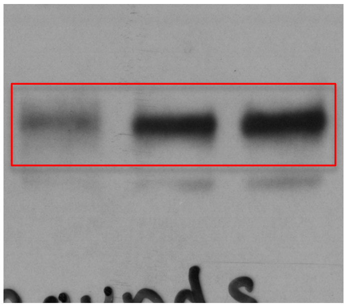

95 kD
72 kD

Lane 1 DSB bound fraction.
Lane 2 DSB bound fraction plus CK2i 50 uM.
Lane 4 DSB bound fraction plus CK2i 100 uM.
Probed for MRE11.

L1 L2 L3

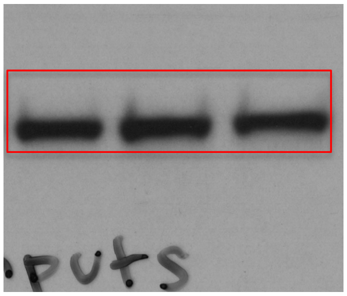

95 kD
72 kD

Lane 1 Total extract.
Lane 2 Total extract plus CK2i 50 uM.
Lane 4 Total extract plus CK2i 100 uM.
Probed forMRE.

L1 L2 L3

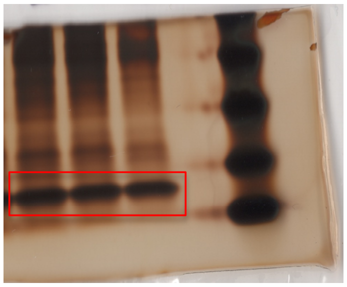

17 kD
10 kD

Lane 1 DSB bound fraction.
Lane 2 DSB bound fraction plus CK2i 50 uM.
Lane 4 DSB bound fraction plus CK2i 100 uM.
Stained with silver..

Fig 7E

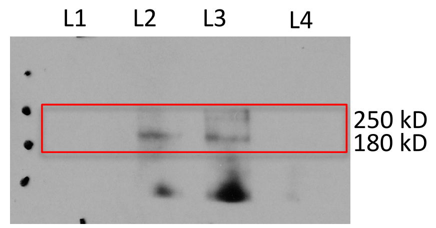

Lane 1 GST bound fraction.
Lane 2 GST-MDC1 bound fraction.
Lane 3 GST-MDC1 bound fraction plus CK2i 50 uM.
Lane 4 GST-MDC1 bound fraction plus CK2i 100 uM.
Probed for TOPBP1.

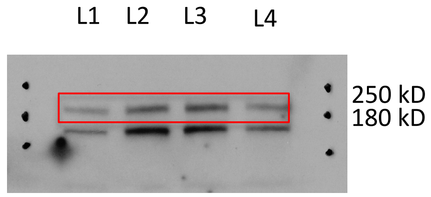

Lane 1 Total extract plus GST.
Lane 2 Total extract plus GST-MDC1 .
Lane 3 Total extract plus GST-MDC1 and CK2i 50 uM.
Lane 4 Total extract plus GST-MDC1 and CK2i 100 uM.
Probed for TOPBP1.

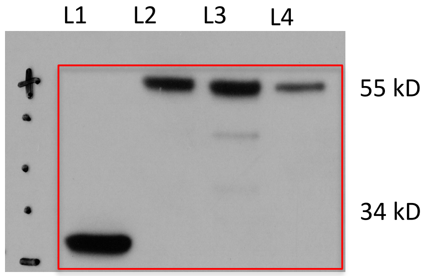

Lane 1 GST bound fraction.
Lane 2 GST-MDC1 bound fraction.
Lane 3 GST-MDC1 bound fraction plus CK2i 50 uM.
Lane 4 GST-MDC1 bound fraction plus CK2i 100 uM.
Probed for GST.
